# Diversity of Fungi Associated with Diseases of Cultivated Brassicaceae in Southern Italy

**DOI:** 10.3390/jof12010013

**Published:** 2025-12-24

**Authors:** Marwa Mourou, Maria Luisa Raimondo, Milan Spetik, Francesco Lops, Gaetana Ricciardi, Maria Grazia Morea, Ales Eichmeier, Antonia Carlucci

**Affiliations:** 1 Department of Agricultural Sciences, Food, Natural Resources and Engineering (DAFNE), University of Foggia, Via Napoli 25, 71122 Foggia, Italy; marwa.mourou@unifg.it (M.M.); francesco.lops@unifg.it (F.L.); gaetana.ricciardi@unifg.it (G.R.); maria.morea@unifg.it (M.G.M.); 2 Mendeleum–Institute of Genetics, Faculty of Horticulture, Mendel University in Brno, Valticka 334, 691 44 Lednice, Czech Republic; milan.spetik@gmail.com (M.S.); ales.eichmeier@mendelu.cz (A.E.)

**Keywords:** Brassica disease, *Alternaria*, *Fusarium*, *Plectosphaerella*, *Stemphylium*, *Sclerotinia* species, MSP-PCR, Multilocus sequence analysis

## Abstract

This study investigated the fungal species associated with symptomatic cultivated Brassica crops in Apulia, Southern Italy, during the 2022–2023 growing seasons. Twenty-two samples from *Brassica oleracea* var. *botrytis*, *B. oleracea* var. *italica*, and *B. rapa* var. *cymosa* showing stunting, wilting, necrotic spots, and lesions were analyzed using morphological and molecular analyses. A total of 259 fungal isolates were obtained, mainly belonging to the genera *Alternaria*, *Plectosphaerella*, *Fusarium*, and *Sclerotinia*, with *Alternaria* and *Plectosphaerella* being the most frequent. Microsatellite PCR (MSP-PCR) profiling revealed considerable genetic diversity within the *Alternaria* and *Plectosphaerella* genera, whereas *Fusarium* and *Sclerotinia* showed uniform profiles. Multilocus analyses (ITS, *tef-1α*, *rpb2*, Alt-a1, and *gapdh*) identified nine species as *Alternaria alternata*, *A. brassicicola*, *A. japonica*, *Fusarium solani* species complex, *Plectosphaerella cucumerina*, *P. pauciseptata*, *P. plurivora*, *Sclerotinia sclerotiorum*, and *Stemphylium vesicarium*. While *Alternaria*, *Fusarium*, and *Sclerotinia* species are well-known Brassicaceae pathogens, *P. pauciseptata*, *P. plurivora*, and *S. vesicarium* have been detected here for the first time on cultivated Brassica crops worldwide. These findings highlight significant intraspecific diversity among the detected fungi and expand the current knowledge of fungal diversity associated with symptomatic cultivated Brassica plants.

## 1. Introduction

The *Brassica* genus is the most significant among the 51 genera within the Brassicaceae family. It includes 37 species [1], many of which have edible leaves, roots, seeds, and stems, providing essential nutrients like vitamins, minerals, and dietary fiber [2]. These crops are also recommended for crop rotation and sustainable farming systems because they improve soil health and reduce reliance on chemical fertilizers [3]. The *Brassica* genus is native to the Mediterranean and Saharan regions, where the climate features consist of mild winters followed by hot and dry summers. However, many species within the genus have adapted well to colder growing conditions. Most of the 37 *Brassica* species are annual or biennial plants, ranging from weedy wild species to domesticated crops [4]. The most widely cultivated *Brassica* species include *B. oleracea* (e.g., cabbage, cauliflower, broccoli), *B. rapa* (e.g., turnip, Pak choi), and *B. napus* (e.g., canola, rapeseed) [5]. Worldwide, Brassicaceae crops are grown in a wide range of climates, from temperate to tropical areas, due to their adaptability and resilience. The leading countries in the production of Brassicaceae are China, India, the United States, and several European countries, each region focusing on different species based on local conditions and agricultural practices [5].

The economic weight of Brassicaceae in Italian agriculture is considerable. According to 2024 Istat data, the market value of Brassicaceae continues to rise due to increased yield efficiency and high demand. Apulia and Sicily have seen an expansion in Brassicaceae production, driven by both domestic consumption and growing export markets. These regions enjoy favorable climatic conditions, which enable year-round production and strengthen Italy’s position in European vegetable exports [6].

In particular, Apulia showed an increase in the cultivation of Brassica crops, especially in terms of the utilized agricultural area (UAA) and production values. The area dedicated to the cultivation of Brassicaceae, such as cabbage, cauliflower, and turnip tops, increased from 3850 hectares in 2023 to 4090 hectares in 2024, and in the same timespan, the yield increased from 23 to 28 tons per hectare [6]. Apulia is also one of the leading regions in Italy for the export of Brassica crops, with a substantial portion of the export to European markets significantly contributing to its own agricultural economy. Additionally, Apulia’s specialized crops, such as “broccoli di Cima di Rapa” (an ecotype of broccoli), are in high demand for both domestic consumption and international trade, further strengthening the region’s role in global vegetable markets [6].

Despite their agricultural importance, Brassica vegetables are susceptible to several severe diseases that need careful monitoring in order to avoid a significant impact on yield and quality [7]. Among the fungal diseases reported worldwide on cultivated *Brassica* crops are the dark leaf spot caused by *Alternaria* spp. [8,9,10,11,12,13], the anthracnose caused by *Colletotrichum dematium* [14], the powdery mildew caused by *Erysiphe cruciferarum* [7], the fusarium wilt caused by *Fusarium* spp. [15,16,17,18,19], the blackleg or phoma disease caused by *Leptosphaeria maculans* (syn. *Phoma lingam*) [20], the white leaf spot caused by *Neopseudocercosporella capsellae* [21,22,23], the root rot caused by *Plectosphaerella cucumerina* [24,25], the wirestem caused by *Rhizoctonia solani* [26,27,28], and the white mold disease caused by *Sclerotinia sclerotiorum* [29,30,31].

Among the most dangerous fungal species causing disease symptoms and severe impacts on yield and quality on cultivated *Brassica* plants, *Alternaria*, *Fusarium*, *Leptosphaeria*, *Neopseudocercosporella*, and *Sclerotinia* species are recognized as highly severe pathogens affecting all brassicaceous plants and especially cultivated ones.

The *Alternaria* genus is a group of fungi belonging to the Ascomycetes class that encompasses significant plant pathogens and saprophytes and commonly causes allergies in humans [32,33]. Currently, this genus is classified under Pleosporaceae, within the order Pleosporales (Dothideomycetes class) [34,35], and consists of over 360 recognized species, clustered into 29 sections. Its main host range includes various *Brassica* species such as cabbage (*B. oleracea* var. *capitata*), Chinese cabbage (*B. campestris* var. *chinensis*), cauliflower (*B. oleracea* var. *botrytis*), broccoli (*B. oleracea* var. *italica*), canola (*B. napus*), mustard (*B. juncea*), and wild-grown plants. Several reports confirm their occurrence worldwide across countries such as China [8], Ecuador [12], Italy [10], Kazakhstan [13], Japan [11], Papua New Guinea [36], Russia [37], and the USA [9]. Four species of *Alternaria* are mainly pathogenic to *Brassica* species, such as *A. brassicae* and *A. brassicicola*, followed by *A. japonica* and *A. alternata* [38]. *Alternaria brassicae* is more commonly associated with *B. rapa* and *B. napus,* while *A. brassicicola* is more commonly found on *B. oleracea* [39], which generally requires warmer conditions and a longer period for infection [40].

Fusarium yellows, commonly known as Fusarium wilt, is one of the most devastating diseases affecting cabbage and other crucifers. It results in significant losses in agricultural yield. Currently, this genus is classified under Nectriaceae, within the order Hypocreales (Sordariomycetes class), comprising over 80 globally distributed species, which exhibit a broad host range and rapid growth, leading to significant challenges to the production of canola (*Brassica napus*) and other crops [41]. Among *Fusarium* species, *F. avenaceum* [19], *F. equiseti* [15,18], *F. oxysporum* f. sp. *conglutinans* [16], *F. oxysporum* f. sp. *raphani* [17], *F. solani* species complex [42], and *F. verticillioides* [43] are the most abundantly reported on cultivated *Brassica*.

One of the most economically serious diseases of Brassica crops, particularly on oilseed rape, is the Blackleg disease, also known as Phoma stem canker, caused by the *L. maculans* species complex [44,45,46]. This disease is globally spread, with increased severity in regions such as Europe, Australia, and North America, which are characterized by high summer temperatures [47]. It was observed on the leaf, flower, and corymb of several *Brassica* species, such as turnip, Chinese cabbage, pak choi, oilseed rape, swede, and mustard. In the UK, Phoma stem canker of oilseed rape is the most economically important disease in Southern, Eastern, and Central England. Severe losses can occur in cauliflower and swede, but it is mainly a leaf blemish on other vegetable brassicas.

When leaf spots appear, the fungus remains latent until severe stem symptoms become visible. Moreover, this fungus can remain viable in the soil for many years after the initial disease report. Despite the introduction of cultivars with enhanced resistance, this pathogen still has a substantial economic impact [48].

*Neopseudocercosporella capsellae* (white leaf spot disease) represents a major threat to cruciferous crops, causing substantial yield losses under cool and wet conditions [49]. Recently, the prevalence of this pathogen has globally increased, and it is now recognized as a re-emerging disease affecting oilseed rape and oriental Brassica vegetables, particularly in the UK [50], the USA [51,52], and Australia [53,54]. This species exhibits a broad host range, including both wild and cultivated crucifers (vegetable brassicas and forage crops) [49].

Lastly, *Sclerotinia sclerotiorum* is a fungal species classified under the Sclerotiniaceae, within the order Helotiales (Leotiomycetes class), that has a host range exceeding 400 species [55]. In the Brassicaceae family, it is reported as the most important pathogen causing disease on the *B. napus* in China. This pathogen is abundantly widespread in Europe and North America too, especially in favourable moist conditions where it causes a soft stem rot. Over the last 10 years, this fungal species has been reported on cabbage [29,31] and broccoli too [30]. As it is the case with *L. maculans*, the pathogen can remain viable in the soil for many years after the initial disease report via the production of sclerotia. The pathogen is not only restricted due to causing problems in open fields but also affects postharvest conditions [40].

In Italy, few reports are available of fungal diseases on cultivated Brassicaceae. In particular, Siciliano et al. [56] reported five *Alternaria* species, namely, *A. alternata*, *A. tenuissima*, *A. arborescens*, *A. brassicicola*, and *A. japonica* on cabbage, cauliflower, wild (*Diplotaxis tenuifolia*), and cultivated rocket (*Eruca sativa*). Other reports only regarded wild and cultivated rockets. Indeed, Garibaldi et al. [24,57] reported *A. japonica* and, for the first time, *P. cucumerina* on cultivated rocket. Lastly, Gilardi et al. [58] reported, for the first time in Italy, *Fusarium equiseti* and *Rhizoctonia solani* as pathogens of wild and cultivated rocket.

Due to the increasing production and the utilized agricultural area (UAA) for the cultivation of Brassicaceae, and also taking into account the lack of available information on the spread of the fungal diseases, a detailed survey was carried out in Northern Apulia from 2022 to 2023 in several Brassica fields. The survey focused on some *Brassica* varieties, such as *B. oleracea* var. *botrytis* (cauliflower), *B. oleracea* var. *italica* (broccoli), *B. oleracea* var. *italica* (mugnoli), and *B. rapa* var. *cymosa* (turnip), showing different symptoms on stems, leaves, and corymbs. Therefore, it aimed to characterize fungal diversity associated with symptomatic cultivated *Brassica* plants by morphological and molecular tools.

## 2. Materials and Methods

### 2.1. Sampling and Fungal Isolation

During the 2022–2023 growing seasons, 22 symptomatic samples consisting of stems, leaves, and corymbs were collected from distinct Brassicaceae species (4 samples of *B. oleracea* var. *botrytis,* 13 samples of *B. oleracea* var. *italica*, and 5 samples of *B. rapa* var. *cymosa*) from cultivated crops in three different areas of Foggia province, Apulia (Southern Italy) (Table S1).

For each sample collected, the disease incidence (DI) and disease severity (DS) were assessed (Table S1). The DI was calculated as a proportion of a number of plants showing symptoms divided by the total number of plants observed according to the following formula:

$$DI\;(\%)=\left(\frac{Number\;of\;diseased\;plants}{Total\;no.\;of\;plants\;observed}\right)\times 100$$

The disease severity was calculated, using a pathometric scale of 0–5, where 0 = no symptoms observed; 1 = 1–20%; 2 = 21–40%; 3 = 41–60%; 4 = 61–80%; and 5 = 81–100% of tissue surface showing symptoms. The overall disease severity (DS) was calculated according to the following formula:

$$DS=\left(\frac{N.\;of\;infected\;plants\;\times \;values\;of\;scores}{Total\;no.\;of\;cases}\right)$$

**Figure 1:**
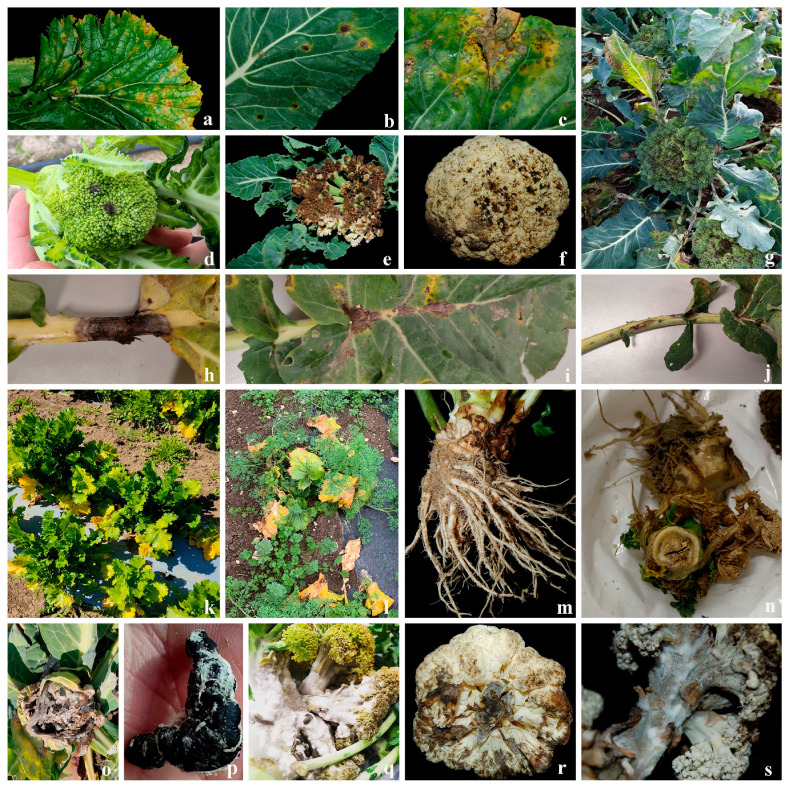
Symptoms observed on cultivated *Brassica* crops. (**a**–**c**) Concentric spots on leaves with chlorotic halo; (**d**–**g**) irregular dark spots on corymb; (**h**–**j**) elongated dark brown/black lesions on main leaf vein; (**k**,**l**) leaf yellowing; (**m**,**n**) collar and root rot; (**o**–**s**) water-soaked lesions with white fluffy mycelium and black sclerotia on corymb.

The symptoms observed consisted of plant stunting and wilting, leaf yellowing, and necrotic concentric spots, with or without a chlorotic halo, irregular dark spots on the corymb, elongated dark brown/black lesions on the stem, water-soaked lesions with white fluffy mycelia, and black sclerotia on the corymb (Figure 1).

Subsequently, after disinfection, the samples were transported to the laboratory for mycological analyses, according to the protocol of Fisher et al. [59], by lowering the percentage of the sodium hypochlorite solution from 5% to 2%. Five small tissue portions were placed on a potato dextrose agar (PDA, Sigma-Aldrich, Milan) medium supplemented with 500 mg L^−1^ of streptomycin sulphate (Oxoid Ltd.) and incubated for 7 days at 25 ± 3 °C. The morphological and culture features were preliminarily used to attribute the fungal genera [60,61,62,63]. All fungal colonies morphologically similar to *Alternaria*, *Fusarium*, *Plectosphaerella*, and *Sclerotinia* species were grown until they sporulated; then, a conidial suspension or hyphal tips were spread on water agar (WA) plates. After 24–36 h of incubation, single germinated propagules were transferred to fresh plates of PDA. The isolation frequency (IF; %) per Brassicaceae cultivar was calculated as the number of tissue portions infected by a given fungus divided by the subtotal number of tissue segments incubated and expressed as percentages:

$$IF=\left(\frac{N.\;of\;infected\;tissues\;by\;fungus}{Total\;N.\;of\;tissues\;analyzed}\right)\times 100$$

The single-spore (monoconidial) isolates are maintained in the culture collection of the Department of Agricultural Sciences, Food, Natural Resources, and Engineering (DAFNE) of the University of Foggia, Italy.

### 2.2. DNA Extraction

From the above-described isolation technique, 259 fungal strains were collected and preliminarily attributed to different taxa on the basis of morphological features. A selection of 142 fungal strains attributed to four of the most numerous taxa collected, that is to say, *Alternaria* (53), *Plectosphaerella* (47), *Fusarium* (25), and *Sclerotinia* (17) genera, was subjected to further analyses. These latter strains, grown on PDA for 7 days, were subjected to genomic DNA extraction according to Carlucci et al. [64].

### 2.3. MSP-PCR Analysis and Molecular Characterization

All fungal strains (142) of each above-mentioned genus were separately screened by the M13 minisatellite primer (5′-GAGGGTGGCGGTTCT-3′) [65]. The (MSP)-PCR profiles were generated according to Santos and Phillips [66]. The DNA banding patterns of *Alternaria*, *Plectosphaerella*, *Fusarium*, and *Sclerotinia* strains were analyzed through the BIONUMERICS v.5.1 software (Applied Maths, Sint-Martens-Latem, Belgium) by calculating Pearson’s correlation coefficient, and the unweighted pair group method analysis (UPGMA) was calculated by arithmetic means. The reproducibility levels were calculated by comparing the banding profiles obtained for the M13 primer. To this purpose, 10% of the strains were chosen at random from all clusters, and their profiles were analyzed again.

Based on MSP-PCR profiles, representative strains of the *Alternaria* (27), *Plectosphaerella* (15), *Fusarium* (5), and *Sclerotinia* (5) groups were chosen for further molecular characterization and phylogenetic analysis.

The internal transcribed spacer (ITS) of the ribosomal DNA region of 52 fungal strains (27 *Alternaria*, 15 *Plectosphaerella*, 5 *Fusarium*, and 5 *Sclerotinia*) was amplified by PCR through the universal primers ITS1 and ITS4, according to White et al. [67]. Based on ITS sequence analysis, 23 strains were identified as belonging to the *Alternaria* genus. These strains were subjected to the amplification of fragments from three genes: the translation elongation factor 1-alpha (*tef-1α*), the Alternaria major allergen gene (Alt-a1), and the second largest subunit of RNA polymerase II (*rpb2*). The respective primers used for the amplification were EF1-728F/EF1-986R as described by Carbone and Kohn [68], Dir5cAlta1–Inv4Alta1 as described by Pavòn et al. [69], and fRPB2-5f2/fRPB2-7c, as described by Sung et al. [70] (Table 1). The remaining four strains within the *Alternaria* group were identified as belonging to the *Stemphylium* genus. These strains were subjected to the amplification of glyceraldehyde-3-phosphate dehydrogenase (*gapdh*) using the primers gpd1/gpd2 according to Berbee et al. [71] (Table 1).

For the *Plectosphaerella* strains, fragments of translation elongation factor 1-alpha (*tef-1α*) and RNA polymerase II’s second largest subunit (*rpb2*) genes were also amplified by means of primer sets EF1-983F/EF1-2218R and RPB2-5F2/RPB2-7cR according to Giraldo et al. [72] (Table 1).

For the *Fusarium* strains, a fragment of the translation elongation factor 1-alpha gene (*tef-1α*) was amplified through the primer set EF1/EF2 according to O’Donnell et al. [73] (Table 1).

The PCR amplifications were performed utilizing G2 Flexi DNA polymerase (Promega, Madison, WI, USA), thus following the protocols described in Table 1. The resulting products were purified by means of NucleoSpin Gel and the PCR Clean-up Kit (Macherey-Nagel, Düren, Germany), following the manufacturer’s protocol. Subsequently, the purified products were sequenced from both ends by using the Sanger method at Eurofins Genomics (Ebersberg, Germany).

**Table 1:** Primers and PCR conditions used in this study.

Locus	Primer	Primer DNA Sequence 5′→3′	PCR Conditions	Reference
ITS	ITS1	TCCGTAGGTGAACCTGCGG	94 °C–3 min; [94 °C–30 s, 55 °C–30 s, 72 °C–30 s] × 35; 72 °C–10 min.	[67]
	ITS4	GCTGCGTTCTT ATCGATGC		
*tef-1α*	EF1-728F	CATCGAGAAGTTCGAGAAGG	94 °C–3 min; [94 °C–30 s, 58 °C–30 s, 72 °C–30 s] × 35; 72 °C–10 min.	[68]
	EF1-986R	TACTTGAAGGAACCCTTACC		
	EF-983F	GCYCCYGGHCAYCGTGAYTTYA	94 °C–5 min; [94 °C–45 s, 54 °C–45 s, 72 °C–60 s] × 36; 72 °C–10 min.	[74]
	EF-2218R	ATGACACCRACRGCRACRGTYT		
	EF1	ATGGGTAAGGARGACAAGAC	94 °C–3 min; [94 °C–60 s, 53 °C–60 s, 72 °C–120 s] × 35; 72 °C–10 min.	[73]
	EF2	GGARGTACCAGTSATCATGTT		
Alt-a1	Dir5cAlta1	GAGAACAGCTTCATGGACTTCTCTTT	94 °C–1 min; [94 °C–30 s, 55–30 s, 72 °C–45 s] × 35; 72 °C–5 min.	[69]
	Inv4Alta1	CGCGGCAGTAGTTGGGAA		
*rpb2*	fRPB2-5f2	GAYGAYMGWGATCAYTTYGG	94 °C–2.3 min; [94 °C–60 s, 50–53 °C–30 s, 72 °C–120 s] × 35; 72 °C–10 min.	[70]
	fRPB2-7cR	CCCATRGCTTGYTTRCCCAT		
*gapdh*	gpd1	CAACGGCTTCGGTCGCATTG	96 °C for 2.00 min, followed by 30 cycles of denaturation at 96 °C for 60 s, annealing at 48 °C for 1 min, and elongation at 72 °C for 45 s. With each cycle, the time at 72 °C was extended by 4 s. Usually, the products from the first amplification were purified and then reamplified with the same primers using another 25 PCR cycles like the initial 30 but with 54 °C of annealing temperature.	[71]
	gpd2	GCCAAGCAGTTGGTTGTGC		

### 2.4. Phylogenetic Analyses

Newly generated DNA sequences, together with those retrieved from GenBank (Table 2 and Table S2), were subjected to phylogenetic analyses in order to identify the *Alternaria* strains. The dataset of each gene was aligned separately by using the MAFFT v. 7, and the alignment obtained was manually checked and edited, when necessary, using Geneious Prime^®^ (v.2023.0.1., Biomatters Ltd., Auckland, New Zealand). A concatenated dataset was built in Sequence Matrix v.1.8 [75], and the missing information sites were denoted by a question mark. The combined dataset (ITS, *tef-1α*, Alt-a1, and *rpb2*) was subjected to maximum likelihood (ML) and Bayesian inference analyses (BI).

Maximum likelihood analysis was performed using IQ-TREE 2 software (Minh et al., 2020 [76]), running 1000 bootstrap replicates. The best-fit evolutionary model for each locus was calculated automatically by IQ-TREE 2 according to the Akaike Information Criterion (AIC). The BI analyses were performed by MrBayes v. 3.2.7 [77,78]. The analyses included four parallel runs of 50 million generations starting from a random tree topology, every 1000 generations were sampled, and the first 25% of the trees were discarded as the “burn-in”. The most suitable substitution model for the BI analyses was determined separately for each locus using jModelTest version 2.1.7 [79]. The trees were visualized in iTOL v. 6.7 [80] and edited in Adobe Illustrator CC 2019. The resulting trees of both methods shared a similar topology; thus, we decided to present ML trees with support values of both methods—bootstrap (BS) and posterior probabilities (pp)—labelled at the nodes.

The consensus sequences of the ITS and the *gapdh* obtained, together with those retrieved from GenBank (Table 2 and Table S2), were subjected to phylogenetic analyses in order to identify the *Stemphylium* strains. All *Stemphylium* sequences were manually concatenated and aligned using MAFFT v.7 (http://mafft.cbrc.jp/alignment/server/, accessed on 26 March 2025) [81]. The alignments were visually checked and manually improved where necessary. Multigenic analyses, according to maximum likelihood and maximum parsimony, were carried out for the ITS and the *gapdh* genes of the *Stemphylium* sequence data. The maximum likelihood analysis was performed using IQ-TREE 2 software [76], running 1000 bootstrap replicates. The best-fit evolutionary model for each locus was calculated automatically by IQ-TREE 2 according to the Akaike Information Criterion (AIC). The maximum parsimony analyses were performed using PAUP, version 4.0b10 [82], through the heuristic search option with 100 random taxa additions, and tree bisection and reconstruction were used as the branch swapping algorithm. Branches of zero length were collapsed, and all multiple equally parsimonious trees were saved. Bootstrap support values were calculated from 1000 heuristic search replicates and ten random taxon additions. The tree length (TL), the consistency index (CI), the retention index (RI), the homoplasy index (HI), and the rescaled consistency index (RC) were calculated for each of them, and the resulting trees were visualized in TreeView, version 1.6.6 [83]. Alignment gaps were treated as missing data. The final trees were selected among the suboptimal trees from each run by comparing the likelihood and bootstrap scores. *Alternaria abundans* (CBS 534.83) and *A. breviramosa* (CBS 121331) were used as outgroups in the multigenic analysis.

Newly generated DNA sequences, together with those retrieved from GenBank (Table 2 and Table S2), were subjected to phylogenetic analyses in order to identify the *Plectosphaerella* strains. All *Plectosphaerella* sequences were manually concatenated and aligned using MAFFT v.7 (http://mafft.cbrc.jp/alignment/server/, accessed on 30 July 2025) [81]. The alignments were visually checked and manually improved, where necessary. Multigenic analyses of the ITS, *tef-1α*, and *rpb2* genes of the *Plectosphaerella* sequence data were carried out as described for the *Stemphylium* strains. *Gibellulopsis serrae* and *G. fusca* were used as outgroups in the multigenic analysis.

In order to identify the *Fusarium* strains, the consensus sequences of ITS and *tef-1α* obtained were exported as a fasta file for the BLASTn analysis. Two web-accessible DNA sequence databases recommended for conducting BLASTn queries to identify that *Fusarium* spp. were used: FUSARIOID-ID by the Westerdijk Fungal Biodiversity Institute, which is publicly available through a web browser (https://www.fusarium.org/page/ID, accessed on 19 June 2025), and the non-redundant NCBI nucleotide collection (i.e., GenBank + EMBL + DDBJ + PDB + RefSeq sequences; http://blast.ncbi.nlm.nih.gov/Blast.cgi, accessed on 19 June 2025) [84].

Lastly, in order to identify the *Sclerotinia* strains, the consensus sequences of ITS were exported as a fasta file for use in the BLASTn analysis. The consensus sequence was compared with those available in the GenBank database using the Basic Local Alignment Search Tool (BLAST, http://www.ncbi.nlm.nih.gov/, accessed on 24 March 2025) to confirm the preliminary morphological identification and ascertain sequence similarity searches.

## 3. Results

### 3.1. Fungal Isolation

The data related to the surveys carried out on 22 samples of Brassicaceae from two different Apulian provinces are summarized in Table 3. A total of 282 strains were collected; 23 of them were attributed to opportunistic bacteria, while the other 259 strains, by preliminary morphological characterization, were identified as the representatives of different fungi. The *Alternaria* group (number of strains [N] = 53; IF = 16.0%) and the *Plectosphaerella* group (N = 47; IF = 14.2%) were the most frequently isolated fungi, followed by the *Fusarium* group (N = 25; IF = 7.6%) and the *Sclerotinia* group (N = 17; IF = 5.2%). Other fungal species, such as *Aspergillus* spp., *Epicoccum* spp., and *Penicillium* spp., were isolated at IFs from 10.0% to 14.5%.

### 3.2. Microsatellite PCR Profiles and Molecular Characterization

**Figure 2:**
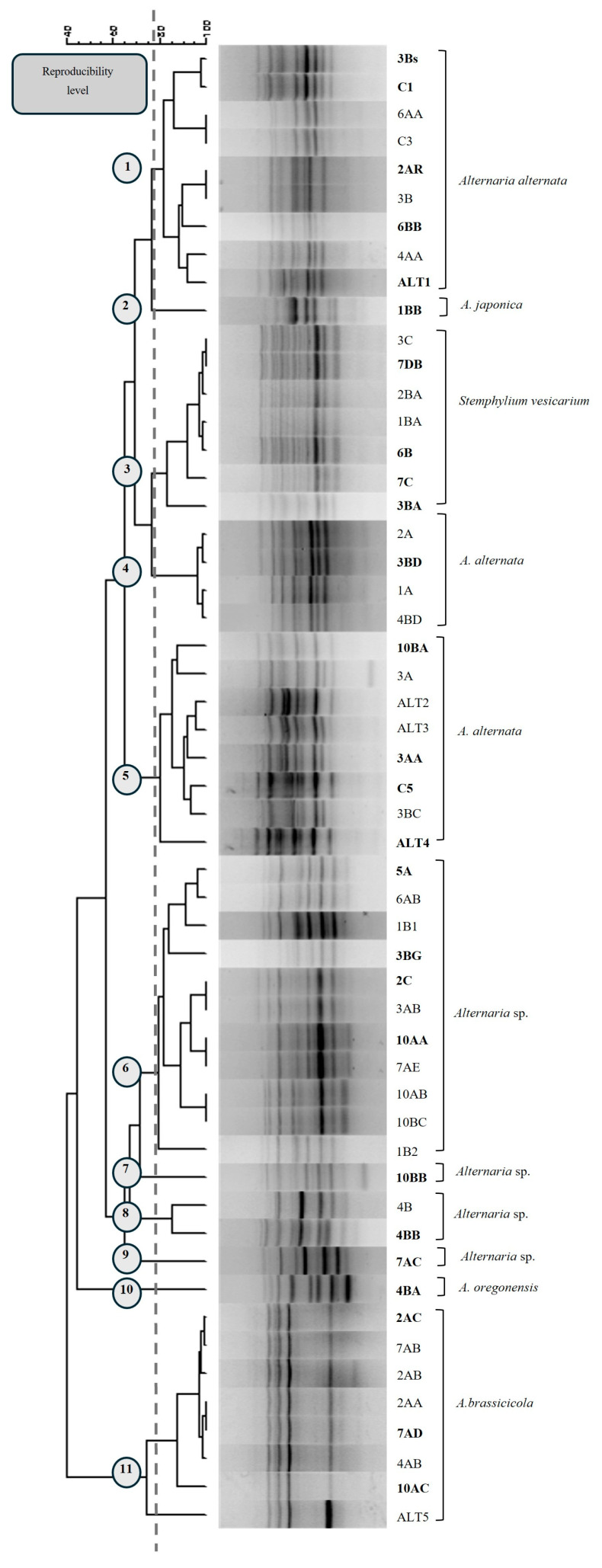
Consensus cladogram from MSP-PCR profiles obtained for *Alternaria* strains (53) with primer M13. The vertical dashed line corresponds to the reproducibility level from which eleven groups of isolates are inferred (indicated by numbered circles).

**Figure 3:**
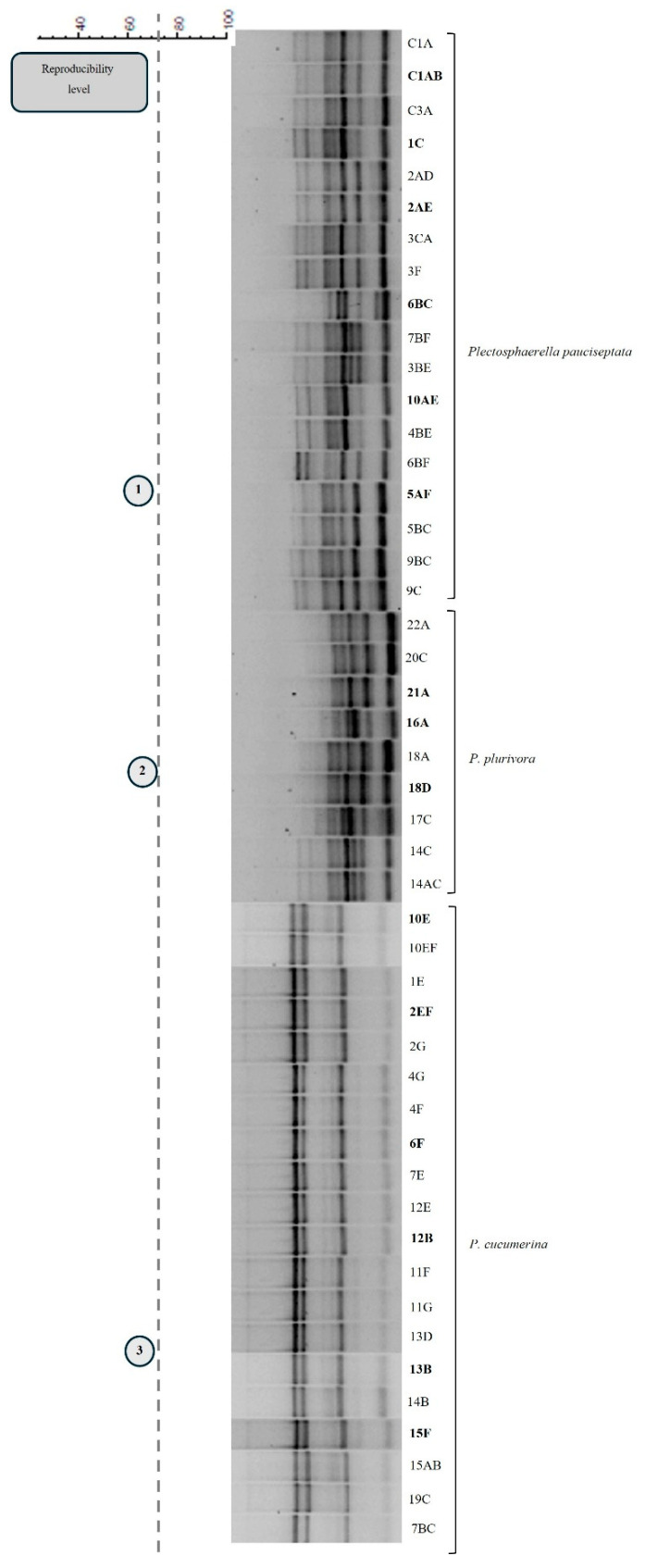
Consensus cladogram from MSP-PCR profiles obtained for *Pletosphaerella* strains (47) with primer M13. The vertical dashed line corresponds to the reproducibility level from which three groups of isolates are inferred (indicated by numbered circles).

Microsatellite PCR profiles were analyzed to select the representative isolates for subsequent molecular analyses. The MSP-PCR dendrogram of the 53 strains belonging to the *Alternaria* group revealed 11 clades, with a reproducibility level of 78% (Figure 2), allowing the identification of distinct groups from which representative strains were chosen. The MSP-PCR dendrogram of the 47 *Plectosphaerella* strains revealed three clades, with a reproducibility level of 73% (Figure 3), providing a tool for selecting strains showing different fingerprint diversity. Lastly, the MSP-PCR analysis of the 25 *Fusarium* and the 17 *Sclerotinia* strains revealed only one microsatellite pattern profile for each group; therefore, the MSP-PCR dendrogram was not generated for these two groups. Specifically, a subset of 27 representative strains of the *Alternaria* group, a subset of 15 representative strains of the *Plectosphaerella* group, a subset of five representative strains of the *Fusarium* group, and a subset of five representative strains of the *Sclerotinia* group were chosen for in-depth molecular characterization and phylogenetic analysis.

### 3.3. Molecular Identification of Representative Isolated Fungi

The ITS, *tef-1α*, Alt-a1, and *rpb2* sequences were obtained for the 23 *Alternaria* strains selected from the MSP-PCR profiles, and these were aligned with sequences retrieved from GenBank. The dataset consisted of sequences from 148 taxa, which included the outgroup taxa *Stemphylium herbarum* (CBS 191.86).

After the alignment and exclusion of incomplete portions at either end, the dataset consisted of 1841 characters, including alignment gaps. Among these, 1123 were constant, and 172 were variable and parsimony-uninformative. The detailed results for each individual gene dataset, along with the corresponding models used, can be found in Table 4.

**Figure 4:**
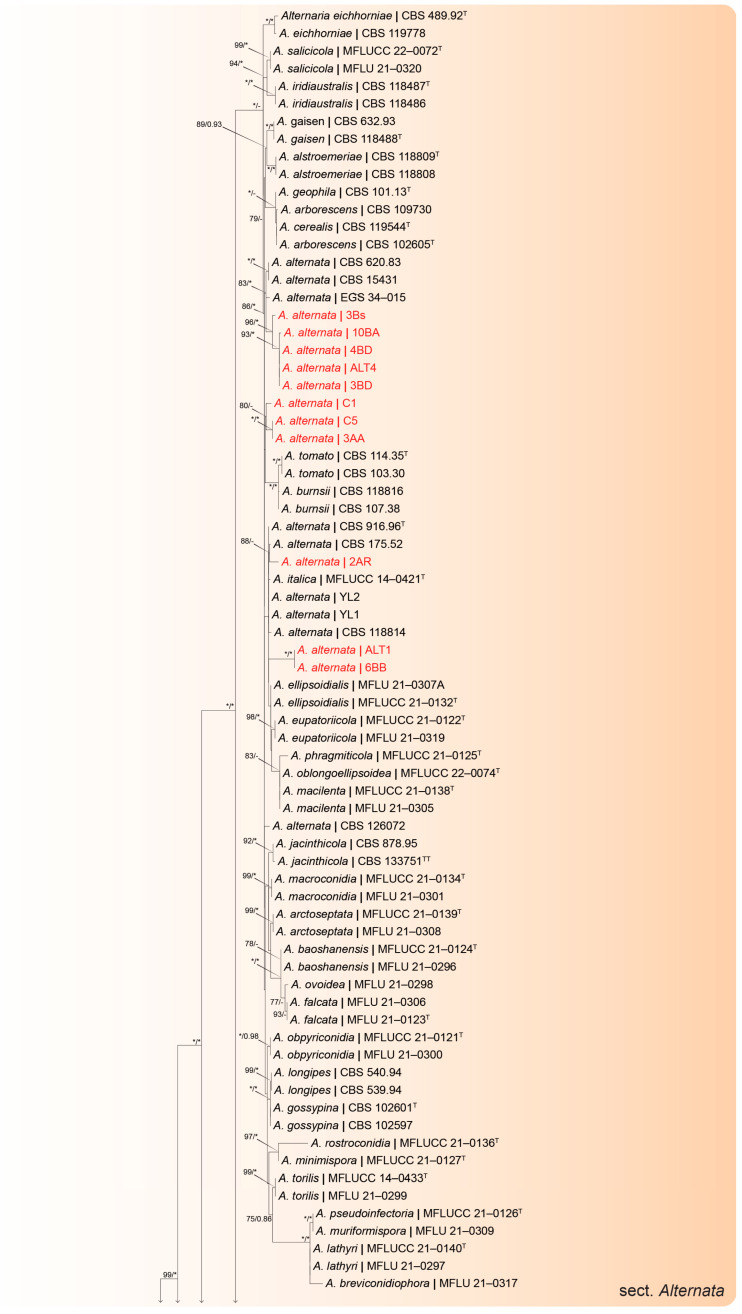

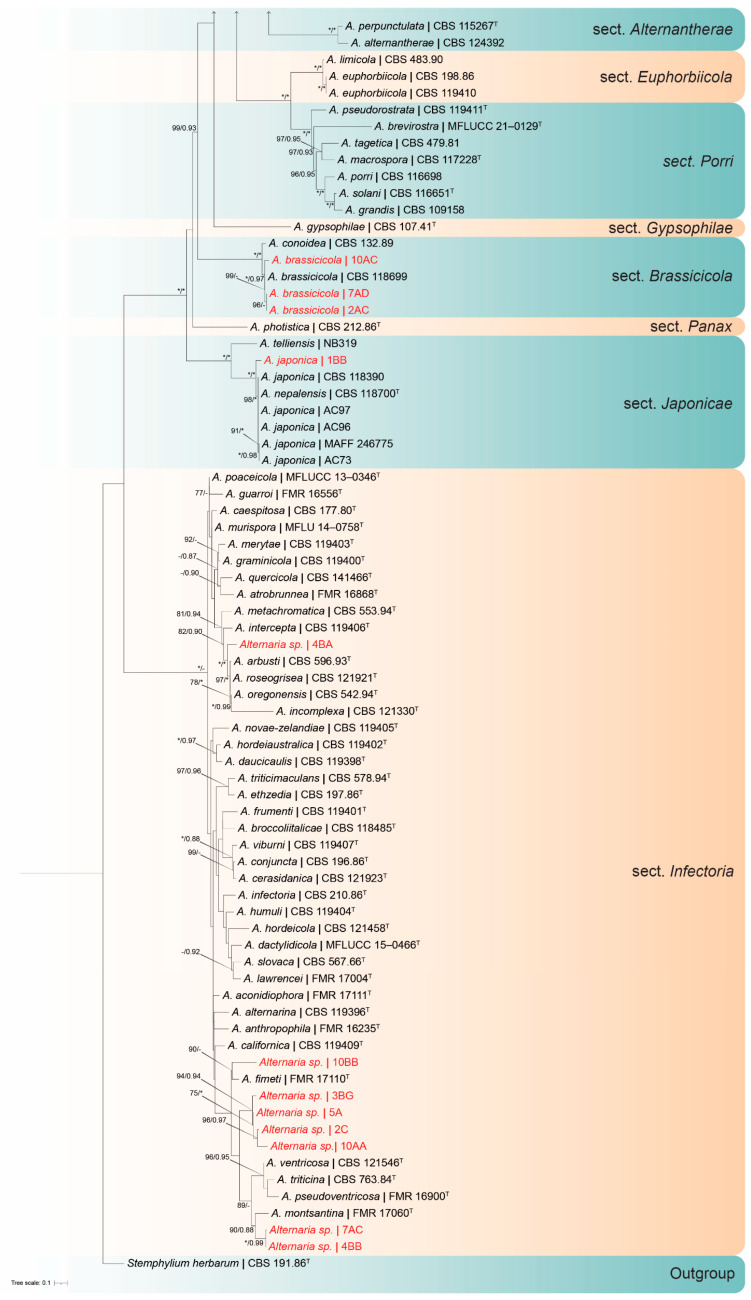
Maximum likelihood tree generated from the combined (ITS, *tef 1-α*, *Alt-a1*, and *rbp2*) *Alternaria* dataset. Support values of both methods bootstrap (BS) and posterior probabilities (pp) labelled at the nodes. Values below 75% (BS) and 0.85 (pp) support are not shown or indicated with a hyphen. Asterisk represents bootstrap values of 100%. Strains obtained in this study are written in red. T indicates *ex-type* strain.

The ML/BI analyses (Figshare: https://figshare.com/s/1ec6360c15ca44c54f54, accessed on 18 July 2025; Figure 4) placed 11 strains in the *Alternaria* section *Alternata*. These 11 strains formed three clusters closely related to reference strains of *A. alternata*. Five strains (3Bs, 10BA, 4BD, ALT4, and 3BD) formed a sister clade to a cluster containing three *A. alternata* strains (CBS 620.83, CBS 15431, and EGS 34-015).

Three strains (2AR, ALT1, and 6BB) were placed among the *ex-type* strain (CBS 916.96) and four other *A. alternata* strains (CBS 175.52, YL2, YL1, and CBS 118814). The remaining three strains (C1, C5, and 3AA) formed a clade between the two aforementioned *A. alternata* clusters. In addition to the strains placed in the *Alternaria* section *Alternata*, three isolates (10AC, 7AD, and 2AC) were displayed in the *Alternaria* section *Brassicola* and formed a fully supported clade with the *ex-type* strain of *A. brassicicola* (CBS 118699). Furthermore, another strain (1BB) was placed in the *Alternaria* section *Japonicae*, clustering with five strains of *A. japonica* (CBS 118390, AC97, AC96, MAFF 246775, and AC73) and the *ex-type* of *A. nepalensis* (CBS118700). Eight strains were identified as *Alternaria* sp. within the *Alternaria* section *Infectoriae*. One strain (4BA) formed a fully supported clade closely related to a cluster containing four known species: *A. arbusti* (CBS 596.91), *A. roseogrisea* (CBS 121921), *A. oregonensis* (CBS 542.94), and *A. incomplexa* (CBS 121330). One strain (10BB) formed a sister branch to the *ex-type* of *A. fimeti* (FMR 17110). Four strains (3BG, 5A, 2C, and 10AA) formed a clade closely related to a cluster containing three *ex-type* strains: *A. ventricosa* (CBS 121546), *A. triticina* (CBS 763.87), and *A. pseudoventricosa* (FMR 17060). Finally, two strains (7AC and 4BB) were placed in a sister clade to the *ex-type* strain of *A. montsantina* (FMR 17060).

**Figure 5:**
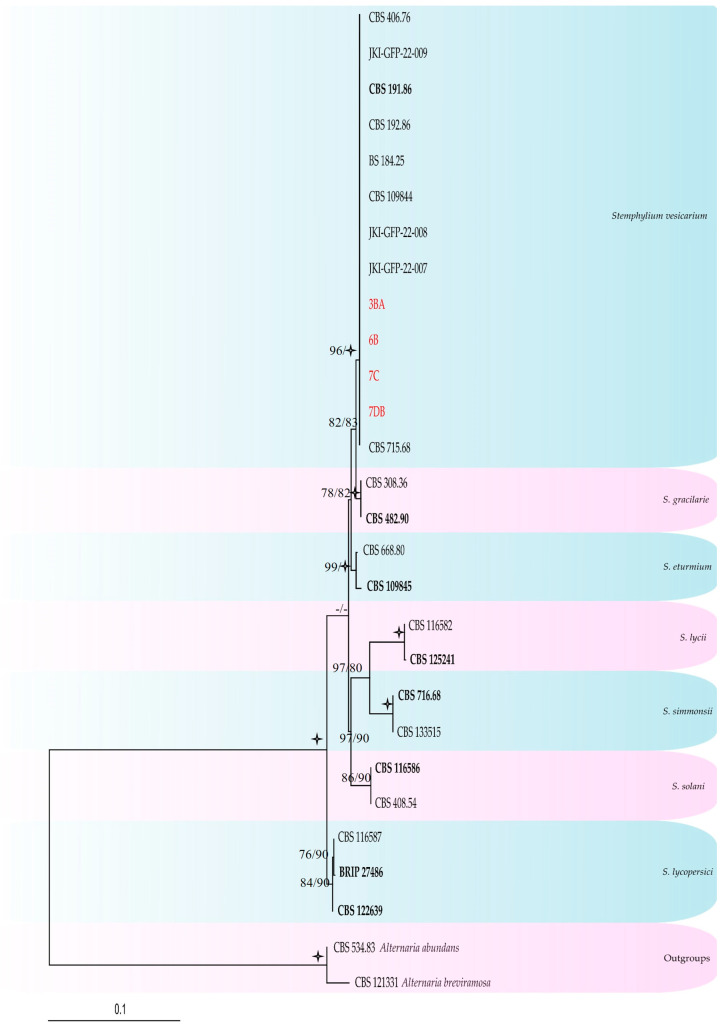
Maximum likelihood tree generated from the combined (ITS, *gapdh*) *Stemphylium* dataset. Bootstrap support values from maximum likelihood/maximum parsimony labelled at the nodes. Values below 75% (BS) support are not shown or indicated with a hyphen. Star symbols represent bootstrap values of 100%. Strains obtained in this study are written in red. *Ex-type* isolates are in bold face.

The ITS and *gapdh* sequences were generated for four *Stemphylium* strains selected from the MSP-PCR profiles, and these were aligned with sequences retrieved from GenBank (Table 2 and Table S2). Detailed results for each individual gene of the combined dataset, along with the corresponding models used, can be found in Table 4 The combined dataset consisted of sequences from 30 taxa, which included the outgroup taxa *Alternaria abundans* (CBS 534.83) and *A. breviramosa* (CBS 121331). After the alignment and exclusion of incomplete portions at either end, the dataset consisted of 1146 characters, including alignment gaps. Among these, 947 were constant, and 17 were variable and parsimony-uninformative. Maximum parsimony analysis of the remaining 182 parsimony-informative characters resulted in four most parsimonious trees (TL = 250; CI = 0.880; RI = 0.925; RC = 0.814; HI = 0.120). Maximum likelihood analysis produced a tree with similar topology (Figshare: https://figshare.com/s/1ec6360c15ca44c54f54, accessed on 18 July 2025; Figure 5). The MP/ML analyses (Figure 5) placed the four *Stemphylium* strains (3BA, 6B, 7C, and 7DB) in a well-supported clade with the *ex-type* sequences of *S. vesicarium* (CBS 191.86).

**Figure 6:**
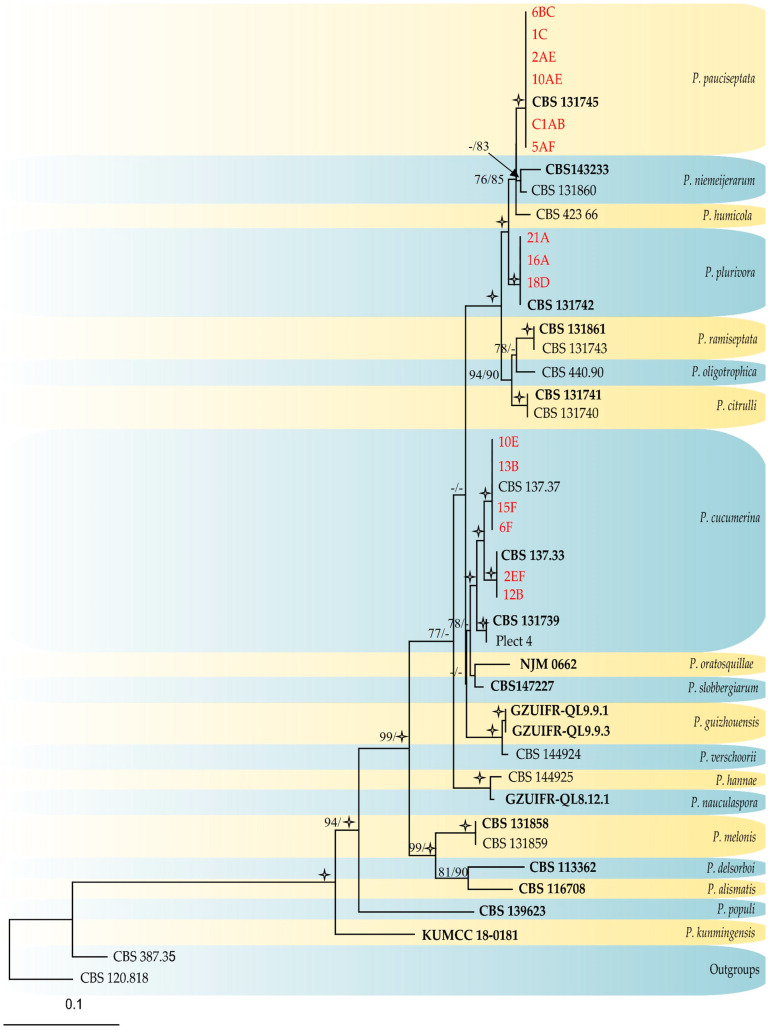
Maximum likelihood tree generated from the combined (ITS, tef-1α and *rpb2*) *Plectosphaerella* dataset. Bootstrap support values from maximum likelihood/maximum parsimony labelled at the nodes. Values below 75% (BS) support are not shown or indicated with a hyphen. Star symbols represent bootstrap values of 100%. Strains obtained in this study are written in red. *Ex-type* isolates are in bold face.

The ITS *tef-1α* and *rpb2* sequences were generated for 15 *Plectosphaerella* strains selected from the MSP-PCR profiles, and these were aligned with sequences retrieved from GenBank (Table 2 and Table S2). The detailed results for each individual gene of the combined dataset, along with the corresponding models used, can be found in Table 4. The combined dataset consisted of sequences from 44 taxa (Table 2 and Table S2), which included the *Gibellulopsis serrae* (CBS 387.35) and *G. fusca* (CBS 120.818) outgroup taxa. After the alignment and exclusion of incomplete portions at either end, the dataset consisted of 2562 characters, including alignment gaps. Among these, 1918 were constant, and 179 were variable and parsimony-uninformative. Maximum parsimony analysis of the remaining 465 parsimony-informative characters resulted in one most parsimonious tree (TL = 1629; CI = 0.558; RI = 0.769; RC = 0.429; HI = 0.442). Maximum likelihood analysis produced a tree with similar topology (Figshare: https://figshare.com/s/1ec6360c15ca44c54f54, accessed on 18 July 2025; Figure 6). The MP/ML analyses (Figure 6) placed six strains (1C, 2AE, 5AF, 6BC, 10AE, and C1AB) in a clade with the *ex-type* strain of *P. pauciseptata* (CBS 131745), three strains (16A, 18D, and 21A) in a well-supported clade with the *ex-type* strain of *P. plurivora* (CBS 131742), and four strains (6F, 10E, 13B, and 15F) and two other strains (2EF, 12B) in two sister well-supported clades with the *ex-type* of *P. cucumerina* (CBS 137.37) and the *neotype* of *P. cucumerina* (CBS 137.33).

The ITS and *tef-1α* sequences of the five *Fusarium* strains (1F, 3F, 4FG, 5E, and 7GA), analyzed in GenBank by the BLAST tool and by the FUSARIOID-ID database, showed the following similarity rates from 99.65% to 99.84% with several reference strains belonging to the *Fusarium solani* species complex (FSSC) (syn. *Neocosmopora solani*) (CBS 112101; NRRL 32737; NRRL 44924; NRRL 46643; CBS 111722; CBS 117149, NRRL 32484; NRRL 53511).

The ITS sequences of the five *Sclerotinia* strains (2F, 5CD, 13F, 16D, and 18E) analyzed in GenBank by the BLAST tool showed a similarity rate of 100% with the Acc. numbers of *S. sclerotiorum* (OR527163, OR527175, OP164567, KY073614, ON506022, OR527174, KY073612, ON830716, MK828201, KT369007, MH137960, KX184720, and ON506026).

On the basis of the data obtained from MSP-PCR and phylogenetic analyses, it was possible to tentatively assign the percentages of isolation for each of the above-mentioned species in terms of isolates in total. In particular, the species belonging to the *Alternaria* group tentatively consisted of *Alternaria alternata* (N = 21; IF = 6.4%), *A. brassicicola* (N = 8; IF = 2.4%), *Alternaria* sp. (N = 16; IF = 4.8%), and *Stemphylium vesicarium* (N = 7; IF = 2.1). The species belonging to the *Plectosphaerella* group tentatively consisted of *P. cucumerina* (N = 20; IF = 6.1%), *P. pauciseptata* (N = 18; IF = 5.5%), and *P. plurivora* (N = 9; IF = 2.7%). The species belonging to the *Fusarium* and *Sclerotinia* groups tentatively consisted of *F. solani* species complex (N = 25; IF = 7.6%) and *S. sclerotiorum* (N = 17; IF = 5.2%) species, respectively (Table 3).

## 4. Discussion

The present study investigates the occurrence and distribution of various fungal species associated with symptomatic cultivated *Brassica* plants in Apulia (Southern Italy). Although *Aspergillus*, *Epicoccum*, and *Penicillium* species exhibited higher IFs than *Fusarium* and *Sclerotinia* species, they were not considered associated with the symptoms observed. This observation is consistent with previous reports identifying these genera as predominantly saprophytic fungi [85,86,87].

The use of the MSP-PCR technique to screen the remaining 142 isolates of *Alternaria*, *Plectosphaerella*, *Fusarium*, and *Sclerotinia* species successfully revealed genetic variability among strains isolated from cauliflower, broccoli, turnip, and “mugnoli” plants. This approach grouped the strains into eleven main clades for the *Alternaria*-like strains, three clades for the *Plectosphaerella*-like strains, and a single clade for each of the *Fusarium* and *Sclerotinia* groups, thus permitting us to focus subsequent multigenic analyses on 52 selected representative strains. On the basis of the ITS results of the 52 strains, further analysis by multigenic approaches tailored a specific taxonomic resolution of each fungal taxon.

The phylogenetic analysis of the 27 *Alternaria*-like strains revealed that 23 of them belonged to the *Alternaria* genus; four other strains were identified as members of the *Stemphylium* genus. The 23 *Alternaria* strains clustered into four distinct *Alternaria* sections. The members of the *Alternaria* genus are recognized globally as primary responsible agents for some of the most economically significant diseases in *Brassicaceae* crops [88]. The major pathogenic species commonly reported in cultivated *Brassica* include *A. brassicae* and *A. brassicicola*, followed by *A. raphani* and *A. alternata* [38,89]. These pathogens are known to cause substantial yield losses, particularly in *B. oleracea*, and as a consequence, they have considerable economic importance. Humpherson-Jones [39] further noted a host-specific pattern, *A. brassicae* being more frequently associated with *B. rapa* and *B. napus*, while *A. brassicicola* predominantly affects *B. oleracea*.

In the present study, *A. alternata* emerged as the most frequently isolated species and was consistently recovered from all symptomatic *Brassica* samples examined, including cauliflower (*B. oleracea* var. *botrytis*), broccoli and mugnoli (*B. oleracea* var. *italica*), and turnip (*B. rapa* var. *cymosa*). *Alternaria alternata* has previously been reported in Italy by Siciliano et al. [56] as a responsible agent for foliar spots on cabbage, cauliflower, and both wild and cultivated rocket, alongside *A. arborescens*, *A. brassicicola*, and *A. tenuissima*. Furthermore, Matić et al. [10] identified *A. alternata* as the agent of alternariosis in cabbage, and more recently, Ramirez-Villacís et al. [12] have reported it as responsible for leaf spot on broccoli in Ecuador. *Alternaria alternata* has also been associated with Alternaria leaf spot on canola (*B. napus* var. *napus*) and rapeseed (*B.napus* var. *oleifera*) in Australia and Serbia, as documented by Al-Lami et al. [90] and Blagojević et al. [89], respectively. Therefore, to the best of our knowledge, this is the first study that associates *A. alternata* with symptomatic *B. oleracea* var. *italica* (mugnoli) and *B. rapa* var. *cymosa* (turnip) plants worldwide.

The second largest *Alternaria* species isolated was *A. brassicicola*, which exclusively emerged from *B. oleracea* samples (cauliflower and broccoli) at a lower IF than *A. alternata*. This finding aligns with previous studies that identify *A. brassicicola* as a common and severe pathogen of *B. oleracea*, often co-occurring with *A. brassicae* [8,36,38,89]. These results also underscore the marked susceptibility of cauliflower and broccoli to simultaneous infections by multiple *Alternaria* species.

Interestingly, in the present study, only a single isolate of *A. japonica* was recovered from *B. oleracea* var. *italica* (broccoli). This fungus has previously been reported as a responsible agent for damping-off in Chinese cabbage seedlings (*B. rapa* subsp. *chinensis*) in China [91] and for the black spot disease on turnip (*B. rapa* subsp. *rapa*) in Spain [92]. Additionally, *A. japonica* was identified as the pathogen responsible for the black spot disease on kale (*B. oleracea* var. *sabellica*) in South Carolina, USA [93], and on *Brassica oleracea* var. *italica* in Japan [11]. Among other cultivated brassicas, *A. japonica* has also been reported on cultivated rocket [94,95]. In this case, *A. japonica* has also been detected here for first time associated with symptoms observed on broccoli (*B. oleracea* var. *italica*) in Italy.

The multigenic analysis performed in this study did not enable the species-level identification of eight *Alternaria* strains, which were placed within the *Alternaria* section *Infectoriae* and classified only at the genus level as *Alternaria* sp. Although Hong et al. [96] and Chalbi et al. [97] demonstrated that the *Alt-a1* locus is a reliable marker for *Alternaria* species identification, further molecular analyses are still needed to resolve the taxonomic placement of these eight strains. Further multilocus phylogenetic analyses or whole-genome sequencing will be required to confirm this hypothesis. Previous studies have proposed a multigenic approach based on five protein-coding loci (*act*, Alt-a1, *cam*, *gapdh*, and plasma membrane *ATPase*) to clarify phylogenetic relationships among *Alternaria* species, with the *cam* and plasma membrane *ATPase* genes being identified as particularly informative [96,98]. More recently, whole-genome sequencing has emerged as a powerful tool for resolving species boundaries within *Alternaria* and other taxonomically challenging fungal groups [99].

Among the 11 clades identified through MSP-PCR analysis of the *Alternaria* strains, four strains selected as representatives of subclade 3 were identified as *Stemphylium vesicarium* based on the ITS and *gapdh* sequences. *Stemphylium vesicarium* is a filamentous fungus that is able to infect a wide range of hosts worldwide, including asparagus [100], garlic [101], and onion [102]. In 2009, it was reported in China from *Brassica pekinensis* leaves [63,103]. *Stemphylium vesicarium* has been present since the late 1970s in Italy, where it causes brown spot symptoms on pear leaves and fruits in the Po Valley [104]. To date, no information is available about the pathogenic or non-pathogenic role of *S. vesicarium* on cultivated Brassicaceae crops worldwide. Moreover, limited information is available on the relationship between saprophytic and pathogenic populations of *Stemphylium* spp., as well as on the possible host specificity of pathogenic isolates. For example, the closely related species belonging to the *Alternaria* genus are primarily saprophytic, although some act as opportunistic pathogens on a broad range of crops [100]. Finally, isolates of *S. vesicarium* are known to produce phytotoxic metabolites [105], and the leaf blight symptoms observed on onion and garlic have been linked to toxin production [106]. Four secondary metabolites that appear to be host-specific toxins are produced by *S. vesicarium*: stemphylin, stemphyperylenol, stemphyloxin, and stemphol [107]. These compounds are known to facilitate colonization and symptom development by selectively damaging or killing host tissues, but their effects are limited to susceptible host species [108]. In any case, to date, the above-mentioned compounds from *S. vesicarium* are not listed on the US National Center for Computational Toxicology database [109] as mycotoxins for humans and livestock. In our study, isolates of *S. vesicarium* were collected alongside *Alternaria* species from symptomatic leaf samples. This co-occurrence raises questions about potential interactions between these genera and the specific role of *S. vesicarium* in the disease development. Therefore, further investigations are needed to determine the pathogenic potential of *S. vesicarium* on cultivated *Brassica* species. To the best of our knowledge, this is the first detection of *S. vesicarium* being associated with *B. oleracea* var. *italica* (broccoli and mugnoli) worldwide.

The phylogenetic analysis of the 15 *Plectosphaerella*-like strains revealed that they clustered into three distinct species within the *Plectosphaerella* genus, namely *P. cucumerina*, *P. pauciseptata*, and *P. plurivora*. Over the past three decades, *Plectosphaerella* isolates have been collected from a wide range of plant hosts across different countries, *P. cucumerina* being consistently reported as the prevalent species [110,111]. Despite their widespread occurrence, infections caused by *Plectosphaerella* spp. remain poorly characterized. These fungi have been identified as agents of both wilt [112] and root rot diseases [113], although comprehensive studies on their pathogenic behaviour and host interactions are still limited. In Italy, Raimondo and Carlucci [111,114] isolated several *Plectosphaerella* species from basil, parsley, tomato, and pepper and demonstrated, through pathogenicity tests, that these fungi behave as hemibiotrophs. These fungi are capable of transitioning to necrotrophic behavior, inducing stunting syndrome characterized by root and collar rot, vascular discoloration, and foliar symptoms. *Plectosphaerella pauciseptata* was the second most abundantly recovered species in this study. In Italy, this fungus has previously been associated with watermelon, melon, tomato, and pepper [60,111]; basil and parsley [114]; in Japan with lettuce, coriander, and chervil [115]; and recently in China with tomato [116]. *Plectosphaerella plurivora,* here detected in association with symptomatic brassica plants, has recently been implicated alongside other *Plectosphaerella* species as a cause of root and collar rot in asparagus, watermelon, tomato [60]; basil [114]; and ginseng [117]. The detection of *Plectosphaerella* spp. in Brassicaceae crops presented in this study is particularly significant, considering that recent studies worldwide have rarely documented their presence or pathogenic impact on Brassicaceae. Indeed, *P. cucumerina* has been described as a pathogen on wild rocket (*Diplotaxis tenuifolia*), recorded for the first time in Italy, causing leaf spots in several commercial glasshouses in Northern and Southern Italy [24,58,118]. More recently, *P. cucumerina* has also been reported as responsible for root rot on cabbage, both in China and worldwide [25]. Our findings align with previous reports, as *P. cucumerina* was the most frequently isolated species within the *Plectosphaerella* genus, exhibiting the highest IF values. *Plectosphaerella cucumerina*, *P. pauciseptata*, and *P. plurivora* were isolated from *B. oleracea* and *B. rapa*. In particular, *P. cucumerina* was isolated from symptomatic stems, leaves, and corymbs of cauliflower, broccoli, and turnip, as well as from symptomatic stems and leaves of mugnoli (*B. oleracea* var. *italica*). By contrast, *P. pauciseptata* and *P. plurivora* were consistently isolated from symptomatic stems and leaves of both *B. oleracea* and *B. rapa*. Moreover, all three *Plectosphaerella* species were collected alongside *Fusarium* and *Alternaria* species from symptomatic leaf, stem, and root samples. Also, in this case, co-occurrence raises questions about potential interactions between these genera and the specific role of *Plectosphaerella* species in disease development. These results suggest that the host range of the *Plectosphaerella* genus may encompass a broad spectrum of species within the Brassicaceae family, thus highlighting the importance of further research into its epidemiology, pathogenicity, and host specificity. To the best of our knowledge, this is the first detection of *P. cucumerina* associated with *B. oleracea* var. *italica* (broccoli) in Italy and with *B. oleracea* var. *italica* (mugnoli), *B. oleracea* var. *botrytis* (cauliflower), and *B. rapa* var. *cymosa* (turnip) worldwide. Additionally, this is the first detection of *P. pauciseptata* and *P. plurivora* associated with symptomatic *B. oleracea*, *B. italica*, and *B. rapa* plants worldwide.

Given the limited reports of *Plectosphaerella* species associated with *Brassica* crops, our findings expand the fungal diversity and geographic distribution of the genus in these crops. However, their pathogenic significance cannot be inferred without targeted pathogenicity assays, which will be carried out for this purpose.

Lastly, *F. solani* species complex and *S. sclerotiorum* were also isolated from *Brassica* plants with lower IF than *Alternaria* and *Plectosphaerella*, although their involvement in *Brassica* diseases is already well-documented. Several *Fusarium* species are known to infect *Brassica* crops, including both vascular *Fusarium* species (causing Fusarium wilt and yellows) and parenchymatous species (causing head and root rots). Among the wilt- and yellow-inducing *Fusarium* species, *Fusarium oxysporum* f. sp. *conglutinans* has been reported on canola in Argentina [16]; *F. oxysporum* f. sp. *raphani* on *B. oleracea* in Italy [17]; *F. equiseti* on cabbage and cauliflower in Korea and China [15,17]; and *F. solani* species complex on cauliflower in China [42]. As far as the parenchymatous *Fusarium* species are concerned, *F. verticillioides* has been identified as the agent of head rot in *B. rapa* subsp. *parachinensis* in China [43], while *F. avenaceum* has been reported as an agent of head rot in *B. oleracea* var. *botrytis* in Poland [19]. Our results are in line with Chai et al. [42], because we collected *F. solani* species complex from all *Brassica* samples showing symptoms of wilting and leaf yellowing. These findings reinforce their established pathogenic roles in Brassica diseases. To the best of our knowledge, this is the first study that associated the *F. solani* species complex with symptomatic *B. oleracea* var. *italica* (broccoli and mugnoli) and *B. rapa* var. *cymosa* (turnip) plants worldwide.

*Sclerotinia sclerotiorum* is a well-known and economically significant pathogen of cultivated *Brassica* species, and it is considered the most important pathogen of *B. napus* in China [55]. Over the past decade, *S. sclerotiorum* has been reported as a pathogen of cabbage [29,31] and broccoli [30]. This pathogen is not limited to field-grown *Brassica,* but it also causes post-harvest injuries during transit and storage [40]. In our study, *S. sclerotiorum* was collected from corymbs of cauliflower, broccoli, and turnip, showing water-soaked lesions with white fluffy mycelia and black sclerotia. To the best of our knowledge, this is the first study that associated *S. sclerotiorum* with symptomatic *B. oleracea* var. *botrytis* (cauliflower) and *B. rapa* var. *cymosa* (turnip) plants both in Italy and worldwide.

In conclusion, this study highlights the complexity of the fungal species associated with cultivated *Brassica* crops in Southern Italy and addresses the need to conduct regular and detailed surveys in order to confirm and accurately identify the fungal species involved. The molecular tools revealed substantial intra- and interspecific diversities across several fungal genera. The frequent co-occurrence of multiple fungal taxa from the same symptomatic plants highlights the complexity of fungal diversity associated with cultivated *Brassica* crops and underscores the need for further studies. Moreover, infections on *Brassica* plants by *Alternaria*, *Stemphylium*, and *Fusarium* pathogens are also linked to the production of several mycotoxins, which may pose risks to human health. In light of this, comprehensive pathogenicity studies on newly reported fungal species associated with cultivated *Brassica* crops are essential to: deepen our knowledge; investigate the pathogenicity, ecological, and epidemiological role; clarify the biological significance of the fungal species here detected; and support the development of effective disease management strategies.

## Figures and Tables

**Table 2:** Information on *Alternaria*, *Fusarium*, *Plectosphaerella*, *Sclerotinia*, and *Stemphylium* strains isolated from Brassica crops collected in Foggia province, Italy. Isolate numbers in bold indicate representative isolates selected for molecular characterization.

Species	Isolate Number	Location	Host	Variety	Year	Part of Plant		GenBank Accession Number			
							ITS	*tef1-α*	Alt-a1	*rpb2*	*gapdh*
*Alternaria alternata*	**ALT1**	Lucera	*Brassica oleracea* var. *botrytis*	Trinacria	2022	Leaves	PV872872	PV952645	PV929800	PV934163	-
	ALT2	Lucera	*Brassica oleracea* var. *botrytis*	Trinacria	2022	Leaves	-	-	-	-	-
	ALT3	Lucera	*Brassica oleracea* var. *botrytis*	Trinacria	2022	Leaves	-	-	-	-	-
	**ALT4**	Lucera	*Brassica oleracea* var. *botrytis*	Akinen	2023	Stem	PV872878	PV952651	PV929806	PV934169	-
	**C1**	Lucera	*Brassica oleracea* var. *botrytis*	Akinen	2023	Stem	PV872873	PV952646	PV929801	PV934164	-
	**C5**	Lucera	*Brassica oleracea* var. *botrytis*	Aprilia	2023	Stem	PV872877	PV952650	PV929805	PV934168	-
	**3AA**	Cerignola	*Brassica oleracea* var. *italica*	Parthenon	2022	Leaves	PV872876	PV952649	PV929804	PV934167	-
	C3	Lucera	*Brassica oleracea* var. *italica*	Parthenon	2023	Stem	-	-	-	-	-
	1A	Cerignola	*Brassica oleracea* var. *italica*	Mugnoli	2022	Leaves	-	-	-	-	-
	2A	Cerignola	*Brassica oleracea* var. *italica*	Mugnoli	2022	Leaves	-	-	-	-	-
	**2AR**	Cerignola	*Brassica oleracea* var. *italica*	Mugnoli	2022	Leaves	PV872871	PV952644	PV929799	PV934161	-
	3B	Cerignola	*Brassica oleracea* var. *italica*	Mugnoli	2022	Leaves	-	-	-	-	-
	3BC	Cerignola	*Brassica oleracea* var. *italica*	Mugnoli	2023	Leaves	-	-	-	-	-
	**3BD**	Cerignola	*Brassica oleracea* var. *italica*	Parthenon	2023	Leaves	PV872874	PV952647	PV929802	PV934165	-
	4AA	Cerignola	*Brassica oleracea* var. *italica*	Parthenon	2023	Leaves	-	-	-	-	-
	4BD	Cerignola	*Brassica rapa* var. *cymosa* (Turnip)	Centoventina	2023	Leaves	-	-	-	-	-
	6AA	Foggia	*Brassica rapa* var. *cymosa* (Turnip)	Centoventina	2023	Leaves	-	-	-	-	-
	**6BB**	Cerignola	*Brassica oleracea* var. *italica*	Parthenon	2023	Leaves	PV872870	PV952643	PV929798	PV934162	-
	**10BA**	Cerignola	*Brassica oleracea* var. *italica*	Parthenon	2023	Leaves	PV872875	PV952648	PV929803	PV934166	-
	3A	Cerignola	*Brassica oleracea* var. *italica*	Mugnoli	2022	Leaves	-	-	-	-	-
	**3Bs**	Cerignola	*Brassica rapa* var. *cymosa* (Turnip)	Centoventina	2022	Leaves	PV872869	PV952642	PV929797	PV934160	-
*A. brassicicola*	ALT5	Lucera	*Brassica oleracea* var. *botrytis*	Akinen	2023	Leaves	-	-	-	-	-
	2AA	Cerignola	*Brassica oleracea* var. *italica*	Parthenon	2022	Stem	-	-	-	-	-
	2AB	Cerignola	*Brassica oleracea* var. *italica*	Parthenon	2023	Stem	-	-	-	-	-
	**2AC**	Cerignola	*Brassica oleracea* var. *italica*	Parthenon	2022	Stem	PV920688	PV952661	PV934179	PV942081	-
	4AB	Cerignola	*Brassica oleracea* var. *italica*	Parthenon	2022	Leaves	-	-	-	-	-
	7AB	Cerignola	*Brassica oleracea* var. *italica*	Parthenon	2022	Leaves	-	-	-	-	-
	**7AD**	Cerignola	*Brassica oleracea* var. *italica*	Parthenon	2023	Leaves	PV920689	PV952662	PV934180	PV942082	-
	**10AC**	Cerignola	*Brassica oleracea* var. *italica*	Parthenon	2023	Leaves	PV920690	PV952663	PV934181	PV942083	-
*A. japonica*	**1BB**	Cerignola	*Brassica oleracea* var. *italica*	Parthenon	2023	Stem	PV920679	PV952652	PV934170	PV942072	-
*Alternaria* sp.	1B1	Lucera	*Brassica oleracea* var. *botrytis*	Akinen	2023	Leaves	-	-	-	-	-
	1B2	Lucera	*Brassica oleracea* var. *botrytis*	Akinen	2023	Leaves	-	-	-	-	-
	**2C**	Cerignola	*Brassica oleracea* var. *italica*	Mugnoli	2022	Leaves	PV920684	PV952657	PV934175	PV942077	-
	**5A**	Cerignola	*Brassica oleracea* var. *italica*	Mugnoli	2022	Leaves	PV920680	PV952653	PV934171	PV942073	-
	3AB	Foggia	*Brassica oleracea* var. *italica*	Parthenon	2023	Leaves	-	-	-	-	-
	**4BB**	Cerignola	*Brassica oleracea* var. *italica*	Parthenon	2023	Stem	PV920685	PV952658	PV934176	PV942078	-
	6AB	Cerignola	*Brassica oleracea* var. *italica*	Parthenon	2023	Stem	-	-	-	-	-
	**7AC**	Cerignola	*Brassica oleracea* var. *italica*	Parthenon	2023	Stem	PV920686	PV952659	PV934177	PV942079	-
	7AE	Cerignola	*Brassica oleracea* var. *italica*	Parthenon	2023	Corymb	-	-	-	-	-
	**10AA**	Cerignola	*Brassica oleracea* var. *italica*	Parthenon	2023	Leaves	PV920683	PV952656	PV934174	PV942076	-
	10AB	Cerignola	*Brassica oleracea* var. *italica*	Parthenon	2023	Leaves	-	-	-	-	-
	**10BB**	Cerignola	*Brassica oleracea* var. *italica*	Parthenon	2022	Corymb	PV920682	PV952655	PV934173	PV942075	-
	10BC	Cerignola	*Brassica oleracea* var. *italica*	Parthenon	2022	Leaves	-	-	-	-	-
	**3BG**	Cerignola	*Brassica rapa* var. *cymosa* (Turnip)	Centoventina	2023	Leaves	PV920681	PV952654	PV934172	PV942074	-
	4B	Foggia	*Brassica oleracea* var. *italica*	Parthenon	2023	Leaves	-	-	-	-	-
	**4BA**	Cerignola	*Brassica oleracea* var. *italica*	Parthenon	2022	Leaves	PV920687	PV952660	PV934178	PV942080	-
*Fusarium solani* species complex	1G	Lucera	*Brassica oleracea* var. *botrytis*	Akinen	2022	Stem	-	-	-	-	-
	**1F**	Cerignola	*Brassica oleracea* var. *italica*	Parthenon	2023	Stem	PV920568	PV952664	-	-	-
	1H	Cerignola	*Brassica oleracea* var. *italica*	Parthenon	2023	Stem	-	-	-	-	-
	2G	Cerignola	*Brassica oleracea* var. *italica*	Parthenon	2023	Stem	-	-	-	-	-
	**3F**	Cerignola	*Brassica oleracea* var. *italica*	Parthenon	2023	Stem	-	-	-	-	-
	3GA	Cerignola	*Brassica oleracea* var. *italica*	Parthenon	2023	Stem	-	-	-	-	-
	3H	Cerignola	*Brassica oleracea* var. *italica*	Parthenon	2023	Stem	-	-	-	-	-
	**5E**	Cerignola	*Brassica oleracea* var. *italica*	Mugnoli	2023	Stem	PV920570	PV952666	-	-	-
	6D	Cerignola	*Brassica oleracea* var. *italica*	Mugnoli	2023	Stem	-	-	-	-	-
	6ED	Foggia	*Brassica oleracea* var. *italica*	Parthenon	2022	Leaves	-	-	-	-	-
	**7GA**	Cerignola	*Brassica oleracea* var. *italica*	Mugnoli	2023	Stem	-	-	-	-	-
	7BC	Cerignola	*Brassica oleracea* var. *italica*	Mugnoli	2023	Leaves	-	-	-	-	-
	7D	Cerignola	*Brassica oleracea* var. *italica*	Parthenon	2022	Leaves	-	-	-	-	-
	7F	Cerignola	*Brassica oleracea* var. *italica*	Mugnoli	2023	Leaves	-	-	-	-	-
	9D	Cerignola	*Brassica oleracea* var. *italica*	Parthenon	2022	Leaves	-	-	-	-	-
	9EF	Cerignola	*Brassica oleracea* var. *italica*	Parthenon	2022	Leaves	-	-	-	-	-
	10H	Cerignola	*Brassica oleracea* var. *italica*	Parthenon	2022	Leaves	-	-	-	-	-
	11A	Cerignola	*Brassica oleracea* var. *italica*	Parthenon	2022	Corymb	-	-	-	-	-
	11DE	Foggia	*Brassica oleracea* var. *italica*	Parthenon	2022	Corymb	-	-	-	-	-
	19A	Cerignola	*Brassica oleracea* var. *italica*	Parthenon	2022	Corymb	-	-	-	-	-
	**4FG**	Cerignola	*Brassica rapa* var. *cymosa* (Turnip)	Centoventina	2022	Stem	PV920569	PV952665	-	-	-
	10C	Cerignola	*Brassica rapa* var. *cymosa* (Turnip)	Centoventina	2022	Stem	-	-	-	-	-
	18B	Cerignola	*Brassica rapa* var. *cymosa* (Turnip)	Centoventina	2022	Stem	-	-	-	-	-
	19E	Cerignola	*Brassica rapa* var. *cymosa* (Turnip)	Centoventina	2022	Leaves	-	-	-	-	-
	23A	Cerignola	*Brassica rapa* var. *cymosa* (Turnip)	Centoventina	2022	Leaves	-	-	-	-	-
*Plectosphaerella cucumerina*	4F	Lucera	*Brassica oleracea* var. *botrytis*	Akinen	2023	Corymb	-	-	-	-	-
	**6F**	Lucera	*Brassica oleracea* var. *botrytis*	Akinen	2023	Stem	-	-	-	-	-
	11F	Lucera	*Brassica oleracea* var. *botrytis*	Akinen	2023	Leaves	-	-	-	-	-
	**15F**	Lucera	*Brassica oleracea* var. *botrytis*	Akinen	2023	Leaves	-	-	-	-	-
	19C	Lucera	*Brassica oleracea* var. *botrytis*	Akinen	2023	Leaves	-	-	-	-	-
	2G	Foggia	*Brassica oleracea* var. *italica*	Parthenon	2022	Leaves	-	-	-	-	-
	4G	Cerignola	*Brassica oleracea* var. *italica*	Parthenon	2022	Leaves	-	-	-	-	-
	7BC	Cerignola	*Brassica oleracea* var. *italica*	Mugnoli	2023	Leaves	-	-	-	-	-
	11G	Cerignola	*Brassica oleracea* var. *italica*	Parthenon	2022	Leaves	-	-	-	-	-
	**12B**	Cerignola	*Brassica oleracea* var. *italica*	Parthenon	2022	Leaves	-	-	-	-	-
	**13B**	Cerignola	*Brassica oleracea* var. *italica*	Mugnoli	2023	Leaves	PV920675	PV952639	-	PV929767	-
	14B	Cerignola	*Brassica oleracea* var. *italica*	Mugnoli	2023	Stem	-	-	-	-	-
	15AB	Cerignola	*Brassica oleracea* var. *italica*	Mugnoli	2023	Stem	-	-	-	-	-
	1E	Cerignola	*Brassica rapa* var. *cymosa* (Turnip)	Centoventina	2023	Stem	-	-	-	-	-
	**2EF**	Cerignola	*Brassica rapa* var. *cymosa* (Turnip)	Centoventina	2023	Stem	PV920674	PV952638	-	PV929766	-
	7E	Cerignola	*Brassica rapa* var. *cymosa* (Turnip)	Centoventina	2023	Stem	-	-	-	-	-
	**10E**	Cerignola	*Brassica rapa* var. *cymosa* (Turnip)	Centoventina	2023	Leaves	-	-	-	-	-
	10EF	Cerignola	*Brassica rapa* var. *cymosa* (Turnip)	Centoventina	2023	Leaves	-	-	-	-	-
	12E	Cerignola	*Brassica rapa* var. *cymosa* (Turnip)	Centoventina	2023	Leaves	-	-	-	-	-
	13D	Foggia	*Brassica rapa* var. *cymosa* (Turnip)	Centoventina	2023	Corymb	-	-	-	-	-
*P. pauciseptata*	6BF	Lucera	*Brassica oleracea* var. *botrytis*	Aprilia	2022	Stem	-	-	-	-	-
	9BC	Lucera	*Brassica oleracea* var. *botrytis*	Akinen	2022	Leaves	-	-	-	-	-
	**C1AB**	Lucera	*Brassica oleracea* var. *botrytis*	Akinen	2022	Leaves	-	-	-	-	-
	2AD	Cerignola	*Brassica oleracea* var. *italica*	Mugnoli	2023	Leaves	-	-	-	-	-
	**2AE**	Cerignola	*Brassica oleracea* var. *italica*	Mugnoli	2023	Leaves	-	-	-		-
	3BE	Cerignola	*Brassica oleracea* var. *italica*	Parthenon	2022	Stem	-	-	-	-	-
	3F	Cerignola	*Brassica oleracea* var. *italica*	Parthenon	2022	Stem	-	-	-	-	-
	4BE	Cerignola	*Brassica oleracea* var. *italica*	Mugnoli	2023	Stem	-	-	-	-	-
	**5AF**	Cerignola	*Brassica oleracea* var. *italica*	Mugnoli	2023	Leaves	PV920673	PV952637	-	PV929765	-
	5BC	Cerignola	*Brassica oleracea* var. *italica*	Parthenon	2023	Leaves	-	-	-	-	-
	**6BC**	Cerignola	*Brassica oleracea* var. *italica*	Parthenon	2022	Leaves	-	-	-	-	-
	7BF	Cerignola	*Brassica oleracea* var. *italica*	Parthenon	2022	Leaves	-	-	-	-	-
	**10AE**	Cerignola	*Brassica oleracea* var. *italica*	Parthenon	2023	Leaves	-	-	-	-	-
	C1A	Cerignola	*Brassica oleracea* var. *italica*	Parthenon	2022	Leaves	-	-	-	-	-
	C3A	Cerignola	*Brassica oleracea* var. *italica*	Parthenon	2023	Leaves	-	-	-	-	-
	**1C**	Cerignola	*Brassica rapa* var. *cymosa* (Turnip)	Centoventina	2022	Stem	PV920672	PV952636	-	PV929764	-
	3CA	Cerignola	*Brassica rapa* var. *cymosa* (Turnip)	Centoventina	2022	Leaves	-	-	-	-	-
	9C	Foggia	*Brassica rapa* var. *cymosa* (Turnip)	Centoventina	2022	Leaves	-	-	-	-	-
	**16A**	Cerignola	*Brassica oleracea* var. *botrytis*	Akinen	2022	Stem	PV920676	PV952640		PV929768	
	18A	Lucera	*Brassica oleracea* var. *botrytis*	Akinen	2022	Leaves	-	-	-	-	-
	**21A**	Lucera	*Brassica oleracea* var. *botrytis*	Akinen	2022	Leaves	-	-	-	-	-
*P. plurivora*	14AC	Cerignola	*Brassica oleracea* var. *italica*	Parthenon	2022	Stem	-	-	-	-	-
	14C	Cerignola	*Brassica oleracea* var. *italica*	Mugnoli	2023	Leaves	-	-	-	-	-
	17C	Cerignola	*Brassica oleracea* var. *italica*	Parthenon	2022	Leaves	-	-	-	-	-
	20C	Cerignola	*Brassica oleracea* var. *italica*	Mugnoli	2023	Leaves	-	-	-	-	-
	**18D**	Cerignola	*Brassica rapa* var. *cymosa* (Turnip)	Centoventina	2022	Stem	PV920677	PV952641	-	PV929769	
	22A	Cerignola	*Brassica rapa* var. *cymosa* (Turnip)	Centoventina	2022	Leaves	-	-	-	-	-
*Sclerotinia sclerotiorum*	1C	Cerignola	*Brassica oleracea* var. *botrytis*	Akinen	2022	Corymb	-	-	-	-	-
	3E	Lucera	*Brassica oleracea* var. *botrytis*	Akinen	2022	Corymb	-	-	-	-	-
	6C	Lucera	*Brassica oleracea* var. *botrytis*	Akinen	2022	Corymb	-	-	-	-	-
	12AB	Lucera	*Brassica oleracea* var. *botrytis*	Akinen	2022	Corymb	-	-	-	-	-
	14D	Lucera	*Brassica oleracea* var. *botrytis*	Akinen	2022	Corymb	-	-	-	-	-
	15B	Lucera	*Brassica oleracea* var. *botrytis*	Akinen	2022	Corymb	-	-	-	-	-
	17AC	Lucera	*Brassica oleracea* var. *botrytis*	Akinen	2022	Corymb	-	-	-	-	-
	**18E**	Lucera	*Brassica oleracea* var. *botrytis*	Akinen	2022	Corymb	-	-	-	-	-
	**2F**	Cerignola	*Brassica oleracea* var. *italica*	Parthenon	2022	Corymb	-	-	-	-	-
	3D	Cerignola	*Brassica oleracea* var. *italica*	Parthenon	2022	Corymb	-	-	-	-	-
	4C	Cerignola	*Brassica oleracea* var. *italica*	Parthenon	2022	Corymb	-	-	-	-	-
	11C	Foggia	*Brassica oleracea* var. *italica*	Parthenon	2022	Corymb	-	-	-	-	-
	**13F**	Cerignola	*Brassica oleracea* var. *italica*	Parthenon	2022	Corymb	PV920572	-	-	-	-
	16D	Cerignola	*Brassica oleracea* var. *italica*	Parthenon	2022	Corymb	-	-	-	-	-
	4D	Cerignola	*Brassica rapa* var. *cymosa* (Turnip)	Centoventina	2023	Corymb	-	-	-	-	-
	**5CD**	Cerignola	*Brassica rapa* var. *cymosa* (Turnip)	Centoventina	2023	Corymb	PV920571	-	-	-	-
	7B	Cerignola	*Brassica rapa* var. *cymosa* (Turnip)	Centoventina	2023	Corymb	-	-	-	-	-
*Stemphylium vesicarium*	1BA	Cerignola	*Brassica oleracea* var. *italica*	Parthenon	2022	Leaves	-	-	-	-	-
	2BA	Cerignola	*Brassica oleracea* var. *italica*	Mugnoli	2023	Leaves	-	-	-	-	-
	3C	Lucera	*Brassica oleracea* var. *botrytis*	Akinen	2022	Stem	-	-	-	-	-
	3BA	Foggia	*Brassica oleracea* var. *italica*	Parthenon	2022	Leaves	-	-	-	-	-
	**6B**	Cerignola	*Brassica oleracea* var. *italica*	Mugnoli	2023	Leaves	PV920566	-	-	-	PV942084
	**7C**	Cerignola	*Brassica oleracea* var. *italica*	Parthenon	2022	Stem	PV920567	-	-	-	PV942085
	7DB	Cerignola	*Brassica oleracea* var. *italica*	Parthenon	2022	Stem	-	-	-	-	-

**Table 3:** Fungal species from four common *Brassicaceae* species collected in Northern Apulia.

	No. of Isolates (IF, %)																
Fungal Species Isolated	*Brassica oleracea* var. *botrytis* “Cauliflower” (Sample Number = 4)				*Brassica oleracea* var. *italica* “Broccoli” (n = 8)				*Brassica oleracea* var. *italica* “Mugnoli” (n = 5)				*Brassica rapa* var. *cymosa* “Turnip” (n = 5)				Total (n = 22)
	Stem	Leaf	Corymb	Subtotal	Stem	Leaf	Corymb	Subtotal	Stem	Leaf	Corymb	Subtotal	Stem	Leaf	Corymb	Subtotal	
*A. alternata*	3 (7.5)	3 (7.5)	0 (0.0)	6 (15.0)	1 (0.7)	5 (3.3)	0 (0.0)	6 (4.0)	0 (0.0)	6 (9.0)	0 (0.0)	6 (9.0)	0 (0.0)	3 (4.2)	0 (0.0)	3 (4.2)	**21 (6.4)**
*A. brassicicola*	0 (0.0)	1 (2.5)	0 (0.0)	1 (2.5)	3 (2.0)	4 (2.6)	0 (0.0)	7 (4.6)	0 (0.0)	0 (0.0)	0 (0.0)	0 (0.0)	0 (0.0)	0 (0.0)	0 (0.0)	0 (0.0)	**8 (2.4)**
*A. japonica*	0 (0.0)	0 (0.0)	0 (0.0)	0 (0.0)	1 (0.7)	0 (0.0)	0 (0.0)	1 (0.7)	0 (0.0)	0 (0.0)	0 (0.0)	0 (0.0)	0 (0.0)	0 (0.0)	0 (0.0)	0 (0.0)	**1 (0.3)**
*Alternaria* spp.	0 (0.0)	2 (5.0)	0 (0.0)	2 (5.0)	3 (2.0)	6 (4.0)	2 (1.3)	11 (7.3)	0 (0.0)	2 (3.0)	0 (0.0)	2 (3.0)	0 (0.0)	1 (1.4)	0 (0.0)	1 (1.4)	**16 (4.8)**
*S. vesicarium*	1 (2.5)	0 (0.0)	0 (0.0)	1 (2.5)	2 (1.3)	2 (1.3)	0 (0.0)	4 (2.6)	0 (0.0)	2 (3.0)	0 (0.0)	2 (3.0)	0 (0.0)	0 (0.0)	0 (0.0)	0 (0.0)	**7 (2.1)**
**Subtotal *Alternaria/Stemphylium* spp.**	**4 (10.0)**	**6 (15.0)**	**0 (0.0)**	**10 (25.0)**	**10 (6.7)**	**17 (11.2)**	**2 (1.3)**	**29 (19.2)**	**0 (0.0)**	**10 (15.0)**	**0 (0.0)**	**10 (15.0)**	**0 (0.0)**	**4 (5.6)**	**0 (0.0)**	**4 (5.6)**	**53 (16.0)**
*P. cucumerina*	1 (2.5)	3 (7.5)	1 (2.5)	5 (12.5)	0 (0.0)	4 (2.6)	0 (0.0)	4 (2.6)	2 (3.0)	2 (3.0)	0 (0.0)	4 (6.0)	3 (4.2)	3 (4.2)	1 (1.4)	7 (9.7)	**20 (6.1)**
*P. pauciseptata*	1 (2.5)	2 (5.0)	0 (0.0)	3 (7.5)	2 (1.3)	6 (4.0)	0 (0.0)	8 (5.3)	1 (1.5)	3 (4.5)	0 (0.0)	4 (6.0)	1 (1.4)	2 (2.8)	0 (0.0)	3 (4.2)	**18 (5.5)**
*P. plurivora*	1 (2.5)	2 (5.0)	0 (0.0)	3 (7.5)	1 (0.7)	1 (0.7)	0 (0.0)	2 (1.3)	1 (1.5)	1 (1.5)	0 (0.0)	2 (3.0)	1 (1.4)	1 (1.4)	0 (0.0)	2 (2.8)	**9 (2.7)**
**Subtotal *Plectosphaerella* spp.**	**3 (7.5)**	**7 (17.5)**	**1 (2.5)**	**11 (27.5)**	**3 (2.0)**	**11 (7.3)**	**0 (0.0)**	**14 (9.2)**	**3 (4.5)**	**6 (9.0)**	**0 (0.0)**	**9 (13.5)**	**5 (6.6)**	**6 (7.0)**	**1 (1.4)**	**12 (16.7)**	**47 (14.2)**
*F. solani* species complex	1 (2.5)	0 (0.0)	0 (0.0)	1 (2.5)	6 (4.0)	5 (3.3)	3 (2.0)	14 (9.3)	3 (4.5)	2 (3.0)	0 (0.0)	5 (7.5)	3 (4.2)	2 (2.8)	0 (0.0)	5 (6.9)	**25 (7.6)**
*S. sclerotiorum*	0 (0.0)	0 (0.0)	8 (20.0)	8 (20.0)	0 (0.0)	0 (0.0)	6 (4.0)	6 (4.0)	0 (0.0)	0 (0.0)	0 (0.0)	0 (0.0)	0 (0.0)	0 (0.0)	3 (4.2)	3 (4.2)	**17 (5.2)**
*Aspergillus* spp.	0 (0.0)	1 (2.5)	1 (2.5)	2 (5.0)	5 (3.3)	8 (5.3)	9 (6.0)	22 (14.6)	0 (0.0)	3 (4.5)	3 (4.5)	6 (9.0)	1 (1.4)	3 (4.2)	2 (2.8)	6 (8.3)	**36 (10.9)**
*Epicoccum* spp.	1 (2.5)	1 (2.5)	0 (0.0)	2 (5.0)	3 (2.0)	7 (4.6)	6 (4.0)	16 (10.6)	0 (0.0)	4 (6.0)	3 (4.5)	7 (10.4)	1 (1.4)	5 (6.9)	2 (2.8)	8 (11.1)	**33 (10.0)**
*Penicillium* spp.	0 (0.0)	1 (2.5)	2 (5.0)	3 (7.5)	7 (4.6)	8 (5.3)	11 (7.3)	26 (17.2)	0 (0.0)	4 (6.0)	4 (6.0)	8 (11.9)	2 (2.8)	3 (4.2)	6 (8.3)	11 (15.3)	**48 (14.5)**
**Total fungi**	**9 (22.5)**	**16 (40.0)**	**12 (30.0)**	**37 (92.5)**	**34 (22.5)**	**56 (37.1)**	**37 (24.5)**	**127 (84.1)**	**6 (8.9)**	**29 (43.2)**	**11 (16.5)**	**46 (68.6)**	**12 (16.6)**	**23 (32.0)**	**14 (19.5)**	**49 (68.1)**	**259 (78.5)**
Opportunistic bacteria	1 (2.5)	0 (0.0)	1 (2.5)	2 (5.0)	0 (0.0)	5 (3.3)	2 (1.3)	7 (4.6)	0 (0.0)	6 (9.0)	0 (0.0)	6 (9.0)	3 (4.2)	5 (6.9)	0 (0.0)	8 (11.1)	**23 (7.0)**
No growth	0 (0.0)	0 (0.0)	1 (2.5)	1 (2.5)	2 (1.3)	7 (4.6)	8 (5.3)	17 (11.3)	2 (3.0)	5 (7.5)	8 (11.9)	15 (22.4)	3 (4.2)	5 (6.9)	7 (9.7)	15 (20.8)	**48 (14.5)**
**Total**	**10 (25.0)**	**16 (40.0)**	**14 (35.0)**	**40 (100.0)**	**36 (23.8)**	**68 (45.0)**	**47 (31.1)**	**151 (100.0)**	**8 (11.9)**	**40 (59.7)**	**19 (28.4)**	**67 (100.0)**	**18 (25.0)**	**33 (45.8)**	**21 (29.2)**	**72 (100.0)**	**330 (100.0)**

**Table 4:** Detailed characteristics of individual region/gene and the best model selected for each gene in the multigenic analyses.

	Locus	No. of Sequences	No. of Characters	Parsimony-Informative	Constant	Unique	Model
***Alternaria***	ITS	144	570	97	430	43	GTR + I + G
	*tef-1α*	140	344	117	184	43	SYM + G
	Alt a1	109	147	85	46	16	HKY + I + G
	*rpb2*	146	780	247	463	70	SYM + I + G
***Total***			**1841**	**546**	**1123**	**172**	**-**
***Stemphylium***	ITS	30	564	65	489	10	K2P + I
	*gapdh*	30	582	117	458	7	K2P + G4
***Total***			**1146**	**182**	**947**	**17**	**-**
***Plectosphaerella***	*ITS*	44	529	67	446	16	TN + F + I + G4
	*tef-1α*	44	749	177	526	46	TIM3 + F + I + G4
	*rpb2*	44	1284	221	946	117	TN + F + I + G4
***Total***			**2562**	**465**	**1918**	**179**	**-**

## Data Availability

The newly generated DNA sequences have been uploaded to NCBI; the accession numbers are shown in this article and Supplementary Materials. The original contributions presented in this study are included in this article and Supplementary Materials.

## Supplementary Materials

The following supporting information can be downloaded at https://www.mdpi.com/article/10.3390/jof12010013/s1. Table S1: *Brassica* samples information (host, locality, incidence, and disease severity); Table S2: Information on *Alternaria*, *Plectosphaerella*, and *Stemphylium* sequences used in the multilocus analyses. *Ex-type* strains are highlighted in bold.
