# Correction: Mourou et al. Diversity of Fungi Associated with Diseases of Cultivated Brassicaceae in Southern Italy. *J. Fungi* 2026, *12*, 13

**DOI:** 10.3390/jof12020139

**Published:** 2026-02-13

**Authors:** Marwa Mourou, Maria Luisa Raimondo, Milan Spetik, Francesco Lops, Gaetana Ricciardi, Maria Grazia Morea, Ales Eichmeier, Antonia Carlucci

**Affiliations:** 1 Department of Agricultural Sciences, Food, Natural Resources and Engineering (DAFNE), University of Foggia, Via Napoli 25, 71122 Foggia, Italy; marwa.mourou@unifg.it (M.M.); francesco.lops@unifg.it (F.L.); gaetana.ricciardi@unifg.it (G.R.); maria.morea@unifg.it (M.G.M.); 2 Mendeleum–Institute of Genetics, Faculty of Horticulture, Mendel University in Brno, Valticka 334, 691 44 Lednice, Czech Republicales.eichmeier@mendelu.cz (A.E.)

## Text Correction

There was an error in the original publication [1]. In Section 3.3, the portion that explains the molecular identification of the *Alternaria* strains shown in Figure 4 is missing. We believe that this text was mistakenly removed during the relocation of figures or tables.

A correction has been made to Section 3.3: Molecular Identification of Representative Isolated Fungi. The new 3rd and 4th paragraphs should read as follows:

The ML/BI analyses (Figshare: https://figshare.com/s/1ec6360c15ca44c54f54, accessed on 18 July 2025; Figure 4) placed 11 strains in the *Alternaria* section *Alternata*. These 11 strains formed three clusters closely related to reference strains of *A. alternata*. Five strains (3Bs, 10BA, 4BD, ALT4, and 3BD) formed a sister clade to a cluster containing three *A. alternata* strains (CBS 620.83, CBS 15431, and EGS 34-015).

Three strains (2AR, ALT1, and 6BB) were placed among the *ex-type* strain (CBS 916.96) and four other *A. alternata* strains (CBS 175.52, YL2, YL1, and CBS 118814). The remaining three strains (C1, C5, and 3AA) formed a clade between the two aforementioned *A. alternata* clusters. In addition to the strains placed in the *Alternaria* section *Alternata*, three isolates (10AC, 7AD, and 2AC) were displayed in the *Alternaria* section *Brassicola* and formed a fully supported clade with the *ex-type* strain of *A. brassicicola* (CBS 118699). Furthermore, another strain (1BB) was placed in the *Alternaria* section *Japonicae*, clustering with five strains of *A. japonica* (CBS 118390, AC97, AC96, MAFF 246775, and AC73) and the *ex-type* of *A. nepalensis* (CBS118700). Eight strains were identified as *Alternaria* sp. within the *Alternaria* section *Infectoriae*. One strain (4BA) formed a fully supported clade closely related to a cluster containing four known species: *A. arbusti* (CBS 596.91), *A. roseogrisea* (CBS 121921), *A. oregonensis* (CBS 542.94), and *A. incomplexa* (CBS 121330). One strain (10BB) formed a sister branch to the *ex-type* of *A. fimeti* (FMR 17110). Four strains (3BG, 5A, 2C, and 10AA) formed a clade closely related to a cluster containing three *ex-type* strains: *A. ventricosa* (CBS 121546), *A. triticina* (CBS 763.87), and *A. pseudoventricosa* (FMR 17060). Finally, two strains (7AC and 4BB) were placed in a sister clade to the *ex-type* strain of *A. montsantina* (FMR 17060).

The authors state that the scientific conclusions are unaffected. This correction was approved by the Academic Editor. The original publication has also been updated.

## References

[1] Mourou M., Raimondo M.L., Spetik M., Lops F., Ricciardi G., Morea M.G., Eichmeier A., Carlucci A. (2026). Diversity of Fungi Associated with Diseases of Cultivated Brassicaceae in Southern Italy. J. Fungi.

