# Supplementary material for: Diversity of Fungi Associated with Diseases of Cultivated Brassicaceae in Southern Italy
Source: J Fungi (Basel). 2025 Dec 24;12(1):13. doi: 10.3390/jof12010013 (PMC12843184; doi:10.3390/jof12010013)
Supplement: Supplementary file 1 [file jof-12-00013-s001.zip › jof-4015101-supplementary.pdf]

Table S1. *Brassica* samples information (host, locality, incidence and disease severity).

N. samples	Host	Common name	Variety	Locality	Disease incidence (%)	Disease severity (DS)
1	***B. oleracea* var. *botrytis***	Cauliflower	Trinacria	Lucera	3.5	0.6
2	***B. oleracea* var. *botrytis***	Cauliflower	Akinen	Cerignola	2.6	0.9
3	***B. oleracea* var. *botrytis***	Cauliflower	Akinen	Lucera	4.6	1.1
4	***B. oleracea* var. *botrytis***	Cauliflower	Aprilia	Lucera	1.2	0.7
5	***B. oleracea* var. *italica***	Broccoli	Parthenon	Cerignola	7.9	1.6
6	***B. oleracea* var. *italica***	Broccoli	Parthenon	Cerignola	5.3	0.8
7	***B. oleracea* var. *italica***	Broccoli	Parthenon	Cerignola	13.6	1.1
8	***B. oleracea* var. *italica***	Broccoli	Parthenon	Foggia	15.4	1.5
9	***B. oleracea* var. *italica***	Broccoli	Parthenon	Lucera	9.8	1.9
10	***B. oleracea* var. *italica***	Broccoli	Parthenon	Lucera	11.3	1.7
11	***B. oleracea* var. *italica***	Broccoli	Parthenon	Cerignola	4.6	2.1
12	***B. oleracea* var. *italica***	Broccoli	Parthenon	Cerignola	13.7	1.3
13	***B. oleracea* var. *italica***	Mugnoli	Mugnoli	Cerignola	2.2	0.7
14	***B. oleracea* var. *italica***	Mugnoli	Mugnoli	Cerignola	12.3	0.5
15	***B. oleracea* var. *italica***	Mugnoli	Mugnoli	Cerignola	1.4	1.0
16	***B. oleracea* var. *italica***	Mugnoli	Mugnoli	Cerignola	3.1	0.3
17	***B. oleracea* var. *italica***	Mugnoli	Mugnoli	Cerignola	5.4	1.1
18	***B. rapa* var. *cymosa***	Turnip	Centoventina	Foggia	10.6	1.0
19	***B. rapa* var. *cymosa***	Turnip	Centoventina	Foggia	8.9	0.4
20	***B. rapa* var. *cymosa***	Turnip	Centoventina	Cerignola	6.4	0.7
21	***B. rapa* var. *cymosa***	Turnip	Centoventina	Cerignola	12.9	0.9
22	***B. rapa* var. *cymosa***	Turnip	Centoventina	Cerignola	5.3	1.2

Table S2. Information on *Alternaria*, *Plectosphaerella*, and *Stemphylium* sequences used in the multilocus analyses. *Ex-type* strains are highlighted in bold.

Species	Strain number[a]	Location	Host	GenBank accession number				
				ITS	*tef1-α*	Alt-a1	*rpb2*	*gapdh*
Alternaria alstroemeriae	CBS 118808	USA	*Alstroemeria* sp.	KP124296	KP125071	KP123845	KP124764	-
	**CBS 118809**	Australia	Culture from *holotype* of *A. alstroemeriae*	NR_163686	KP125072	MH084526	KP124765	-
A. alternantherae	CBS 124392	China	*Solanum melongena*	KC584179	KC584633	KP123846	KC584374	-
	CBS 620.83	USA	*Nicotiana tabacum*	KP124315	KP125091	KP123868	KP124783	-
	CBS 118814	USA	*Solanum lycopersicum*	KP124357	KP125133	KP123906	KP124825	-
	CBS 126072	Namibia	Soil	KP124377	KP125155	KP123925	KP124847	-
	EGS 34-015	Namibia	Soil	AF347032	q	KP275690	JQ905181	-
	YL 1	China	*Magnolia denudata*	MK860132	MN072924	q	MN072920	-
	YL 2	China	*Magnolia denudata*	MN044764	MN072925	q	MN072921	-
	CBS 15431	USA	*Staphylea trifolia*	KP124301	KP125076	KP123851	KP124769	-
	CBS 175.52	USA	*Juncus mertensianus*	KC584227	KC584703	KP123857	KC584445	-
A. alternata	**CBS 916.96; EGS 34-016**	India	*Arachis hypogaea*	AF347031	KC584634	AY563301	KC584375	-
A. arborescens	CBS 109730	USA	*Solanum lycopersicum*	KP124399	KP125177	KP123946	KP124869	-
	**CBS 102605; EGS 39-128**	USA	*Lycopersicon esculentum*	AF347033	KC584636	AY563303	KC584377	-
A. arctoseptata	MFLU 21-0308	Italy	*Spartium* sp.	q	OK236609	OK236756	OK236656	-
	**MFLUCC 21-0139**	Italy	Dead standing stem of *Lathyrus* sp.	q	OK236608	OK236755	OK236655	-
A. baoshanensis	**MFLUCC 21-0124; H_50A**	China	*Cucurbita moschata*	MZ622003	OK236612	OK236759	OK236659	-
	MFLUCC 21-0296; H_50B	China	*Cucurbita moschata*	MZ622004	OK236613	OK236760	OK236660	-
A. brassicicola	**CBS 118699; EGS 42.002**	USA	*Brassica oleracea*	JX499031	KC584642	q	KC584383	-
A. breviconidiophora	MFLU 21-0317; JL-2021c; IT3308_B	Italy	*Digitalis* sp.	MZ621998	OK236605	OK236752	OK236652	-
A. brevirostra	**MFLUCC 21-0129; JL-2021d; IT2195_B**	Italy	*Plantago* sp.	MZ622015	OK236619	q	OK236666	-
A. burnsii	**CBS 107.38**	India	*Cuminum cyminum*	KP124420	KP125198	KP123967	KP124889	-
	CBS 118816	India	*Rhizophora mucronata*	KP124423	KP125201	KP123970	KP124892	-
A. cerealis	**CBS 119544**	New Zealand	*Avena sativa*	MH863062	KP125186	KP123955	KP124878	-

A. conoidea	CBS 132.89	Saudi Arabia	*Ricinus communis*	q	KC584711	FJ348228	KC584452	-
A. eichhorniae	**CBS 489.92**	India	*Eichhornia crassipes*	q	KP125204	KP123973	KP124895	-
	CBS 119778	Indonesia	*Eichhornia crassipes*	KP124426	KP125205	q	KP124896	-
A. ellipsoidialis	MFLU 21-0307A; JL-2021e	Italy	*Brassica* sp.	MZ621990	OK236598	OK236744	OK236644	-
	**MFLUCC 21-0132; JL-2021e**	Italy	*Brassica* sp.	MZ621989	OK236596	OK236743	OK236643	-
A. eupatoriicola	**MFLUCC 21-0122; JL-2021f; IT IT3518_A**	Italy	*Eupatorium cannabinum*	MZ621982	OK236589	OK236736	OK236636	-
	MFLU 21-0319; JL-2021f; IT3518_B	Italy	*Eupatorium cannabinum*	MZ621983	OK236590	OK236737	OK236637	-
A. euphorbiicola	CBS 119410	USA	*Euphorbia pulcherrima*	KJ718173	KJ718521	q	KJ718346	-
	CBS 198.86	USA	*Euphorbia pulcherrima*	KJ718172	KJ718520	KJ718686	KJ718345	-
A. falcata	**MFLU 21-0123; JL-2021g; IT2079_A**	Italy	dead standing stem of *Atriplex* sp.	MZ621992	OK236599	OK236746	OK236646	-
	MFLU 21-0306; JL-2021g; IT2079_B	Italy	dead standing stem of *Atriplex* sp.	MZ621993	OK236600	OK236747	OK236647	-
A. gaisen	CBS 632.93	Japan	*Pyrus pyrifolia* cv. *nijiseiki*	KC584197	KC584658	KP123974	KC584399	-
A. geophila	**CBS 101.13**	Switzerland	Peat soil	KP124392	KP125170	KP123940	KP124862	-
A. gossypina	**CBS 102597; E.G.S. 45.114**	USA, Florida	*Minneola tangelo*	MH862797	KP125211	KP123978	KP124902	-
	**CBS 102601**	Colombia	*Minneola tangelo*	MH862801	KP125212	KP123979	KP124903	-
A. grandis	**CBS 109158**	USA	*Solanum tuberosum*	KJ718239	EU130547	JQ646425	KJ718414	-
A. gypsophilae	**CBS 107.41; EGS 07.025; IMI 264349**	Unknown	*Gypsophila elegans*	KC584199	KC584660	KJ718688	KC584401	-
A. iridiaustralis	CBS 118487	Australia	*Iris* sp.	KP124436	KP125215	KP123982	KP124906	-
	**CBS 118486**	Australia	*Iris* sp.	NR_136120	KP125214	KP123981	KP124905	-
A. italica	MFLUCC 14-0421	Italy	*Vitis vinifera*	MG764017	q	q	MG859737	-
A. jacinthicola	CBS 878.95	Mauritius	*Arachis hypogaea*	KP124437	KP125216	KP123983	KP124907	-
	**CBS 133751**	Mali	*Eichhornia crassipe*	KP124438	KP125217	KP123984	KP124908	-
A. japonica	CBS 118390	USA	*Brassica rapa* subsp. *chinensis*	KC584201	KC584663	-	KC584405	-
	AC73	Japan	*Raphanus sativus*	LC440594	LC480220	LC481625	LC476800	-
	MAFF 246775	Japan	*Raphanus sativus*	LC440595	LC480221	LC481626	LC476801	-
	AC96	Japan	*Brassica oleracea* var. *italica*	LC440596	LC480222	LC481627	LC476802	-
	AC97	Japan	*Brassica oleracea* var. *italica*	LC440597	LC480223	LC481628	LC476803	-
A. lathyri	MFLU 21-0297; JL-2021i; IT1640_B	Italy	Dead stem of *Lathyrus* sp.	MZ621975	OK236582	OK236729	OK236629	-

	**MFLUCC 21-0140**	Italy	Dead aerial stem of *Lathyrus* sp.	MZ621974	OK236581	OK236728	OK236628	-
A. limicola	**CBS 483.90[T]**	Mexico	*Citrus aurantiifolia*	KJ718178	KJ718526	JQ646413	KJ718351	-
A. longipes	CBS 540.94; EGS 30-033	USA	*Nicotiana tabacum*	AY278835	KC584667	AY563304	KC584409	-
	CBS 539.94	USA	*Nicotiana tabacum*	KP124441	KP125220	KP123987	KP124911	-
A. macilenta	**MFLUCC 21-0138; JL-2021j; IT2076_A**	Italy	*Scabiosa* sp.	MZ621972	OK236579	OK236726	OK236626	-
	MFLU 21-0305; JL-2021j; IT2076_B	Italy	*Scabiosa* sp.	MZ621973	OK236580	OK236727	OK236627	-
A. macroconidia	MFLU 21-0301; JL-2021k; IT2088_B	Italy	*Lathyrus* sp.	MZ622002	OK236705	OK236758	OK236658	-
A. macrospora	**CBS 117228**	USA	*Gossypium barbadense*	NR_136045	KC584668	KJ718702	KC584410	-
A. minimispora	**MFLUCC 21-0127; JL-2021l; H 14 A**	Thailand	*Citrullus lanatus*	MZ621980	OK236587	OK236734	OK236634	-
A. muriformispora	MFLU 21-0309; JL-2021m; IT2101_B	Italy	*Plantago* sp.	MZ621977	OK236584	OK236731	OK236631	-
A. nepalensis	**CBS 118700**	Nepal	*Brassica* sp.	KC584207	KC584672	q	KC584414	-
A. oblongoellipsoidea	**MFLUCC 22-0074; JL-2021o; IT2102_A**	Italy	*Cichorium* sp.	MZ621967	OK236574	OK236721	OK236621	-
A. obpyriconidia	**MFLUCC 21-0121; JL-2021p; IT1688_A**	Italy	*Fabaceae*	MZ621978	OK236585	OK236732	OK236632	-
	MFLU 21_0300; JL-2021p; IT1688_B	Italy	*Fabaceae*	MZ621979	OK236586	OK236733	OK236633	-
A. ovoidea	**MFLUCC 14_0427; JL-2021r**	Italy	*Dactylis sp.*	MZ622006	OK236615	OK236762	OK236662	-
A. perpunctulata	**CBS 115267**	USA	*Alternanthera philoxeroides*	KC584210	KC584676	JQ905111	KC584418	-
A. phragmiticola	**MFLUCC 21-0125; JL-2021s; IT2630_A**	Italy	*Phragmites* sp.	MZ621994	OK23660	OK236748	OK236648	-
A. porri	CBS 116698	Puerto Rico	*Allium sativum*	q	KC584679	KJ718726	KC584421	-
A. pseudoinfectoria	**MFLUCC 21-0126; JL-2021u; 2181_A**	Italy	Dead branch	MZ621984	OK236685	OK236738	OK236638	-
A. pseudorostrata	**CBS 119411[T]**	USA, California	*Euphorbia pulcherrima*	JN383483	KC584680	AY563295	KC584422	-
A. rostroconidia	**MFLUCC 21-0136; JL-2021v; IT3515_B**	Italy	*Arabis* sp.	MZ621969	OK236576	OK236724	OK236624	-
A. salicicola	**MFLUCC 22-0072; JL-2021w; T504_A**	Russia	*Aster* sp.	MZ621999	OK236606	OK236753	OK236653	-
	MFLU 21-0320; JL-2021w; T504_B	Russia	*Aster* sp.	MZ622000	OK236607	OK236754	OK236654	-
A. solani	**CBS 116651[T]**	USA, California	*Solanum tuberosum,*	KC584217	KC584688	AY563299	KC584430	-
A. tagetica	CBS 479.81	UK, England	*Tagetes erecta,* seed	KC584221	KC584692	KJ718761	KC584434	-
A. telliensis	NB319	Algeria	*Lycopersicum esculentum* leaf	MT013033	MK904548	MK940313	MK904535	-
A. tomato	CBS 103.30	Unknown	*Solanum lycopersicum*	KP124445	KP125224	KP123991	KP124915	-
	CBS 114.35	Unknown	*Solanum lycopersicum*	KP124446	KP125225	KP123992	KP124916	-

A. torilis	MFLU 21-0299; JL-2021x; 1667_B	Italy	*Torilis* sp.	MZ621987	OK236594	OK236741	OK236641	-
	**MFLUCC 14-0433**	Italy	*Torilis* sp.	MZ621988	OK236595	OK236742	OK236642	-
A. aconidiophora	**CBS 145419; FMR 17111**	Spain/Catalonia	Forest leaf litte	NR_166229	q	q	q	-
A. alternarina	**CBS 119396**	USA/Wisconsin	*Avena sativa*	q	LR134367	JQ905113	JQ905199	-
A. anthropophila	**FMR 16235**	Spain/Catalonia	Human subcutaneous Nodule	LR537444	LR537046	q	LR537040	-
A. arbusti	**CBS 596.93**	USA/California	*Pyrus pyrifolia*	MH862447	FJ214902	q	LR134184	-
A. atrobrunnea	**FMR 16868**	Spain/Catalonia	Human ulcerative skin lesion	LR537033	LR537051	q	LR537044	-
A. broccoli-italicae	**CBS 118485**	Italy	*Brassica pekinensis*	KM821536	LR134262	q	LR134194	-
A. caespitosa	CBS 177.80	Spain	*Ybotryomyces caespitosus*	KC584250	KC584752	q	KC584492	-
A. californica	**CBS 119409**	USA	*Triticum aestivum*	NR_136021	KY352500	JQ646373	LR134181	-
A. cerasidanica	CBS 121923	Denmark/near Skaelskor	Fruit of *Prunus avium*	LR135744	LR135745	q	LR135746	-
A. conjuncta	**CBS 196.86**	Switzerland	*Pastinaca sativa*	NR_135929	KC584649	q	KC584390	-
A. dactylidicola	**MFLUCC 15-0466**	Italy	*Dactylis glomerata*	NR_151852	q	q	KY750720	-
A. daucicaulis	**CBS 119398**	USA	*Daucus carota*	NR_136027	LR134241	q	LR134177	-
A. ethzedia	**CBS 197.86**	Switzerland	*Brassica napus*	AY278833	KC584657	AY563284	KC584398	-
A. fimeti	**CBS 145423; FMR 17110**	Spain/Catalonia	Small rodent dung	LR133920	LR133922	q	LR133923	-
A. frumenti	**CBS 119401**	USA	Undetermined Poaceae	NR_136028	LR134370	JQ646378	LR134172	-
A. graminicola	**CBS 119400**	USA	Undetermined Poaceae	NR_136024	LR134249	q	LR134180	-
A. guarroi	**FMR 16556**	Spain/Catalonia	Human ulcerative skin lesion	LR537031	LR537050	q	LR537045	-
A. hordeiaustralica	**CBS 119402**	USA	*Hordeum vulgare*	NR_136018	LR134243	q	LR134179	-
A. hordeicola	**CBS 121458**	Southwest Norway	*Hordeum vulgare*	NR_136019	LR134371	JQ646372	LR134175	-
A. humuli	**CBS 119404**	France/Alsace	*Humulus lupulus*	JQ693652	LR134199	q	LR134174	-
A. incomplexa	**CBS 121330**	USA	*Canal mud*	JQ693658	LR134250	JQ646374	LR134185	-
A. infectoria	**CBS 210.86**	USA	*Triticum aestivum*	NR_131263	KC584662	FN689402	KC584404	-
A. intercepta	**CBS 119406**	Europe	*Viburnum sp.*	NR_135957	FJ214927	JQ646380	LR134170	-
A. lawrencei	**CBS 145425; FMR 17004**	Spain, Catalonia	Goat dung	NR_166227	LR133912	q	LR133911	-
A. merytae	**CBS 119403**	New Zealand	*Meryta sinclairii*	JQ693651	LR134198	q	LR134119	-
A. metachromatica	**CBS 553.94**	South Australia	*Triticum aestivum*	JQ693660	FJ214931	AY563285	JQ905189	-

A. montsantina	**CBS 145426; FMR 17060**	Spain, Catalonia	Unidentified twig	LR133913	LR133919	q	LR133918	-
A. murispora	**MFLU 14-0758**	Germany	Dead stems	NR_137964	q	q	q	-
A. novaezelandiae	**CBS 119405**	New Zealand	Daucus carota; Culture from *holotype* of *A. novae-zelandiae*	JQ693655	LR134197	JQ646379	LR134120	-
A. oregonensis	**CBS 542.94**	USA	*Triticum aestivum*	NR_135935	KC584674	AY563283	KC584416	-
A. photistica	**CBS 212.86**	UK	*Digitalis purpurea*	MH861944	KC584678	q	KC584420	-
A. poaceicola	**MFLUCC 13-0346; ICMP 21560**	Italy	Dead aerial stem of *Dactylis glomerata* L.	KY026587	q	q	KY460971	-
A. pseudoventricosa	**CBS 145428; FMR 16900**	Spain, Catalonia	Horse dund	LR133928	LR133936	q	LR133934	-
A. quercicola	**CBS 141466**	Iran	Leaves of *Quercus brantii*	KX228295	LR134259	q	LR134188	-
A. roseogrisea	**CBS 121921**	USA	*Helianthus annuus*	LR134102	LR134260	q	LR134192	-
A. slovaca	**CBS 567.66**	Slovakia	Culture from *paratype* of *Chmelia slovaca*	KC584226	KC584702	q	KC584444	-
A. triticimaculans	**CBS 578.94**	Argentina	Culture from *holotype* of *A.triticimaculans*	JQ693657	FJ214930	q	LR134183	-
A. triticina	**CBS 763.84**	India	*Triticum aestivum*	AY278834	FJ214942	q	LR134186	-
A. ventricosa	**CBS 121546**	Qatar	Culture from *holotype* of *A. ventricosa*	JQ693649	KY352501	JQ646377	LR134134	-
A. viburni	**CBS 119407**	Europe	Culture from *holotype* of *A. viburni*	JQ693647	LR134200	q	LR134166	-
Stemphylium herbarum	**CBS 191.86**	India	*Medicago sativa*	KC584239	KC584731	q	KC584471	-
Plectospaherella alismatis	**CBS 113362**	The Netherlands	*Alismata plantago-aquatica*	JF780523	LR026489	-	LR026196	-
P. citrulli	CBS 131740; Plect 151	Italy	Root of *Cucumis melo*	LR026795	LR026490	-	-	-
	**CBS 131741; Plect 157**	Italy	Root of *Citrullus lanatus*	LR026796	LR026491	-	LR026197	-
P. cucumerina	CBS 137.33	England	*Nicotiana tabacum*	LR026797	LR026492	-	LR026198	-
	**CBS 137.37**	Italy	Paper	LR026798	LR026493	-	LR026199	-
P. delsorboi	**CBS 116708**	Italy	*Curcuma alismatifolia*	LR026810	LR026505	-	LR026209	-
P. endophytica	**XY YMF 1.04701**	China	*Hydrilla verticillata*	MW024054	MW029607	-	-	-
P. guizhouensis	**GZUIFR-QL9.9.1; CGMCC 3.19658**	China	Soil	MK880441	MK930453	-	MK930460	-
	GZUIFR-QL9.9.3; CGMCC 3.19660[IS]	China	Soil	MK880443	MK930455	-	MK930462	-
P. hannae	**CBS 144925; JW181001**	The Netherlands	Garden soil	LR590201	LR594767	-	q	-
P. humicola	**CBS 423.66**	Zaire	Soil	LR026811	LR026506	-	LR026210	-
P. kunmingensis	**KUMCC 18-0181**	China	Fruiting body of fungus	MK993014	MK993017	-	MK993018	-
P. melonis	**CBS 131858; Plect 211**	Italy	Melon collar	HQ238965	q	-	q	-

	CBS 131859; Plect 228	Italy	Melon root	HQ238967	q	-	q	-
P. nauculaspora	**GZUIFR-QL8.12.1; CGMCC 3.19656**	China	Soil	MK880439	MK930451	-	MK930458	-
P. oligotrophica	CBS 440.90	Brazil	Soil	LR026814	LR026509	-	LR026211	-
P. oratosquillae	**NJM 0662**	Japan	Mantis shrimp (*Oratosquilla oratoria)*	AB425974	q	-	q	-
P. pauciseptata	**CBS 131745; Plect 186**	Italy	Root of *Solanum esculentum*	LR026816	LR026511	-	LR026212	-
P. plurivora	**CBS 131742; Plect 365**	Italy	Apex of *Asparagus officinalis*	LR026829	LR026524	-	LR026219	-
P. populi	**CBS 139623**	Germany	Branch of *Populus nigra*	KR476750	LR026527	-	LR026222	-
P. ramiseptata	**CBS 131861; Plect 403**	Italy	*Solanum esculentum* root	LR026832	LR026530	-	LR026225	-
	CBS 131743	Italy	*Citrullus lanatus* collar	LR026831	LR026529	-	LR026224	-
P. slobbergiarum	**CBS 147227; NL1930002**	Netherlands	Soil	MW883443	MW890114	-	MW890074	-
P. verschoorii	**CBS 144924; JW 13004**	The Netherlands	Garden soil	LR590241	LR594775	-	LR594801	-
P. vigrensis	**CBS 150722**	Poland	Lesaf of *Impatiens noli-tangere*	OR539695	OR540197	-	OR540198	-
Stemphylium eturmium	CBS 668.80	Greece	*Solanum lycopersicum*	KU850540	-	-	-	KU850688
	**CBS 109845; E.G.S. 29.099; IMI 386969**	New Zealand	*Solanum lycopersicum*	KU850541	-	-	-	KU850689
S. gracilariae	**CBS 482.90; E.G.S. 37.073; ATCC 669721**	Israel	*Gracilaria* sp.	KU850549	-	-	-	AF443883
	CBS 308.36; ATCC 10737	USA	*Solanum lycopersicum*	KU850547	-	-	-	KU850695
S. lycii	CBS 116582; E.G.S. 48.089	USA	*Pistacia vera*	KU850599	-	-	-	KU850745
	**CBS 125241; HSAUP 1833**	China	*Lycium chinense*	KU850602	-	-	-	KU850748
S. lycopersici	CBS 116587; E.G.S. 46.001	Dominican Republic	*Solanum lycopersicum*	KU850608	-	-	-	KU850753
	**CBS 122639; HSAUPV0893**	China	*Solanum lycopersicum*	KU850611	-	-	-	KU850756
	**BRIP: 27486; UMSl0010**	Australia	*Solanum lycopersicum; holotype* of *S.rombundicum*	MK336819	-	-	-	MK336865
S. simmonsii	CBS 133515; E.G.S. 30.153	Canada	*Solanum lycopersicum*	KU850636	-	-	-	KU850777
	CBS 716.68; ATCC 18518; IMI 135458;	USA	*Commelina* sp.	KU850632	-	-	-	KU850773
S. solani	**CBS 116586; E.G.S. 41.135**	USA	*Solanum lycopersicum*	KU850627	-	-	-	KU850768
	CBS 408.54; ATCC 11128	USA	*Solanum lycopersicum*	KU850626	-	-	-	KU850767
S. vesicarium	**CBS 192.86; E.G.S. 36.088; IMI 269683**	Australia	*Medicago sativa; neotype of Pleospora pomorum*	KU850568	-	-	-	KU850715
	CBS 191.86; E.G.S. 36.138; IMI 276975	India	*Medicago sativa*	KC584239	-	-	-	AF443884
	JKI-GFP-22-007	North of Lower Saxony	*Solanum lycopersicum*	ON787815	-	-	-	ON787812

	JKI-GFP-22-008	Southwest of Baden-Wurttemberg	*Solanum lycopersicum*	ON787816	-	-	-	ON787813
	JKI-GFP-22-009	Southwest of Baden-Wurttemberg	*Solanum lycopersicum*	ON787817	-	-	-	ON787814
	CBS 184.25	United Kindom	*Malus domestica*	KU850557	-	-	-	KU850704
	CBS 109844; E.G.S. 29.089; IMI 386968	USA	*Solanum lycopersicum; Ex-type* of *Pleospora tomatonis*	KU850572	-	-	-	KU850719
	CBS 715.68; E.G.S. 12.171	Canada	*Pisum sativum*	KU850565	-	-	-	KU850712
	CBS 406.76	Germany	*Solanum lycopersicum*	KU850566	-	-	-	KU850713
Alternaria abundans	CBS 534.83	New Zealand	*Isotype* of *Embellisia abundans* E.G.Simmons	MH861639	-	-	-	KC584154
A. breviramosa	CBS 121331	Australia	*Triticum* sp.	FJ839608	-	-	-	KC584148

ATCC, American Type Culture Collection; CBS, culture collection of the Westerdijk Fungal Biodiversity Institute, Utrecht, The Netherlands; E.G.S.: Personal collection of Dr. E.G. Simmons; MFLU: the Herbarium of Mae Fah Luang University Chiang Rai, Thailand; MFLUCC: Mae Fah Luang University Culture Collection, Chiang Rai, Thailand; AC: Personal collection of JN; FMR: Facultat de Medicina, Universitat Rovira i Virgili, Reus, Spain; ICMP, International Collection of Microorganisms from Plants; IMI, CABI Genetic Resource Collection; MUCL: (Agro)Industrial Fungi and Yeast Collection of the Belgian Co-ordinated Collections of Micro-organisms (Mycothèque de l'Université Catholique de Louvain).
